# In silico construction of a multiepitope Zika virus vaccine using immunoinformatics tools

**DOI:** 10.1038/s41598-021-03990-6

**Published:** 2022-01-07

**Authors:** Ana Clara Barbosa Antonelli, Vinnycius Pereira Almeida, Fernanda Oliveira Feitosa de Castro, Jacyelle Medeiros Silva, Irmtraut Araci Hoffmann Pfrimer, Edecio Cunha-Neto, Andréa Queiroz Maranhão, Marcelo Macedo Brígido, Renato Oliveira Resende, Anamélia Lorenzetti Bocca, Simone Gonçalves Fonseca

**Affiliations:** 1 Department of Bioscience and Technology, Institute of Tropical Pathology and Public Health, Federal University of Goiás, Rua 235 s/n, sala 335, Setor Universitário, Goiânia, GO 74605-050 Brazil; 2 Departament of Master in Environmental Sciences and Health, School of Medical, Pharmaceutical and Biomedical Sciences, Pontifical Catholic University of Goiás, Goiânia, Brazil; 3 Department of Cell Biology, University of Brasília, Brasília, Brazil; 4 Heart Institute (InCor), School of Medicine, University of São Paulo, São Paulo, Brazil; 5 Institute for Investigation in Immunology (iii) - National Institute of Science and Technology (INCT), São Paulo, Brazil

**Keywords:** Peptide vaccines, Infectious diseases, Immunology

## Abstract

Zika virus (ZIKV) is an arbovirus from the Flaviviridae family and Flavivirus genus. Neurological events have been associated with ZIKV-infected individuals, such as Guillain-Barré syndrome, an autoimmune acute neuropathy that causes nerve demyelination and can induce paralysis. With the increase of ZIKV infection incidence in 2015, malformation and microcephaly cases in newborns have grown considerably, which suggested congenital transmission. Therefore, the development of an effective vaccine against ZIKV became an urgent need. Live attenuated vaccines present some theoretical risks for administration in pregnant women. Thus, we developed an in silico multiepitope vaccine against ZIKV. All structural and non-structural proteins were investigated using immunoinformatics tools designed for the prediction of CD4 + and CD8 + T cell epitopes. We selected 13 CD8 + and 12 CD4 + T cell epitopes considering parameters such as binding affinity to HLA class I and II molecules, promiscuity based on the number of different HLA alleles that bind to the epitopes, and immunogenicity. ZIKV Envelope protein domain III (EDIII) was added to the vaccine construct, creating a hybrid protein domain-multiepitope vaccine. Three high scoring continuous and two discontinuous B cell epitopes were found in EDIII. Aiming to increase the candidate vaccine antigenicity even further, we tested secondary and tertiary structures and physicochemical parameters of the vaccine conjugated to four different protein adjuvants: flagellin, 50S ribosomal protein L7/L12, heparin-binding hemagglutinin, or RS09 synthetic peptide. The addition of the flagellin adjuvant increased the vaccine's predicted antigenicity. In silico predictions revealed that the protein is a probable antigen, non-allergenic and predicted to be stable. The vaccine’s average population coverage is estimated to be 87.86%, which indicates it can be administered worldwide. Peripheral Blood Mononuclear Cells (PBMC) of individuals with previous ZIKV infection were tested for cytokine production in response to the pool of CD4 and CD8 ZIKV peptide selected. CD4 + and CD8 + T cells showed significant production of IFN-γ upon stimulation and IL-2 production was also detected by CD8 + T cells, which indicated the potential of our peptides to be recognized by specific T cells and induce immune response. In conclusion, we developed an in silico universal vaccine predicted to induce broad and high-coverage cellular and humoral immune responses against ZIKV, which can be a good candidate for posterior in vivo validation.

## Introduction

Zika virus (ZIKV) infection has been documented in 87 countries globally (by July of 2019)^1^. Brazil is the country with the highest number of ZIKV infection cases reported in the Americas, with the first registered autochthonous case in 2015^2^. The number of suspected and confirmed cases of autochthonous infections in Brazil reached more than 346 thousand people in the period of 2015 and March of 2017^3^. Even though the death rate is low (< 0.003%)^3^, there were 14,558 suspected cases of microcephaly or congenital neurological malformations, from which 2,952 were confirmed^4^. Although the epidemic appeared to be waning after 2017, 31,576 cases were reported in the Americas in 2018, of which 3,589 were confirmed. In 2019, this number decreased to 12,079 reported cases and 3,482 confirmed cases^1^.

The ZIKV genome is composed by a positive-stranded RNA which codes a polyprotein that is subsequently cleaved into 3 structural proteins (Capsid C, prM, Envelope E) and 7 non-structural proteins (NS1, NS2A, NS2B, NS3, NS4A, NS4B, NS5). ZIKV is a mosquito-borne flavivirus^5^ that has increased global attention in recent years due to the severe symptoms in certain populations. Other forms of transmission include blood transfusion, sexual, intrauterine, perinatal and laboratory manipulation. ZIKV-infected individuals are generally asymptomatic but can manifest rash, body itching, fever, headache, myalgia and arthralgia^3^. Abortion^6^, neurological malformation including microcephaly and other anatomical defects in fetus have also been associated with pregnant women being infected with ZIKV in 5–30% of cases^7,8^; in addition, in rare cases, Guillain-Barré syndrome (GBS), a neurodegenerative disease, has been reported after ZIKV infection^9^.

Protection against ZIKV is greatly associated with neutralizing antibodies against structural proteins, such as the Envelope E, particularly against the domain III^10–12^. As this humoral response is dependent on CD4 + T cells, such cell population is also required for complete protection against ZIKV infection, specially Th1 cells^13–15^. This finding was corroborated by Hassert and colleagues, who observed that in absence of CD4 + T cells, there is an increased disease severity in mice and higher viral titer in the nervous tissues^16^. Another important cell population in the control of ZIKV is CD8 + T cells, as demonstrated when IFN receptor-competent mice lacking CD8+ T cells had increased susceptibility to ZIKV pathologies^17^ and transfer of CD8+ T cells from infected Ifnar1-/- mice to naïve mice lead to protection of the central nervous system after ZIKV challenge^18^. A screening of T cell epitopes revealed that the T cell response is directed mostly against structural proteins, such as prM and E, but also include epitopes from NS1, NS2A-B, NS3, NS4A-B and a high number of epitopes from NS5^5^. CD4 + T cells have been observed against all 10 ZIKV proteins, although most are directed towards the capsid and envelope proteins. CD8 + T-cell responses were mostly directed to NS3, NS4B and NS5 proteins^19^. These findings suggest that the correlates of protection in ZIKV infection are mediated by the complex interplay between specific B and T cells, providing insights into new strategies to guide protective immune responses against the infection.

Due to the risks related to the infection, including congenital malformations, prophylaxis against ZIKV infection is still an urgency, and the best answer is vaccinating people in risk areas, mostly susceptible populations. Pregnant women^7^, elderly^20^ and immunocompromised individuals^21^ deserve special attention. Several vaccine types against ZIKV are currently in phase I clinical trials, including DNA vaccines, mRNA-based, inactivated virus, peptide, and recombinant viral vectored vaccines^22^. However, hitherto only one DNA and one mRNA vaccine coding prM-E have succeeded to enter a phase 2 clinical trial, according to WHO vaccine pipeline tracker in April 2020 (https://www.who.int/). The most predominant form of antigens among them are the prM, E and NS1 proteins, but none of the listed vaccines are multiepitope vaccines or directed to other non-structural proteins^23,24^.

Multiepitope vaccines are composed of individual or overlapping epitopes flanked by linkers (small sequences of amino acids) and have attracted scientific interest because they are a safer alternative for pregnant women and other individuals at risk as they contain only portions of the pathogen proteins, and therefore cannot cause infection. Furthermore, they are able to induce long-lasting humoral and cellular immunity, have the potential to include a repertoire of epitopes with potential to bind a high variety of Human Leukocyte Antigens (HLA) types, and therefore a high population coverage^25^. Multiepitope vaccines containing both B and T cells epitopes against Hepatitis C virus (HCV) have been tested in animal models and demonstrated increase in antibodies and specific T cell response^26^, and an HIV vaccine containing multiple CD8 + T cell epitopes has entered clinical trial phase I (NCT00076037)^27^. Furthermore, there are several multiepitope vaccines in clinical trials phase I and II for neoplasia, including melanoma (NCT00580060, NCT00153569, NCT00003362), breast cancer (NCT03793829, NCT03012100, NCT04144023), glioblastoma^28^, prostate (NCT02362464, NCT02362451) and ovarian cancer (NCT02764333) (https://clinicaltrials.gov/). Altogether, the safety profile and the success of multiepitope vaccines in entering clinical trials are encouraging features for a vaccine against ZIKV.

Aiming to develop such vaccine, we performed extensive literature and bioinformatics analysis to select promising ZIKV protective epitopes derived from all structural and non-structural proteins, seeking promiscuous sequences that cover the highest number of type I and type II HLAs. We assembled a novel  in silico chimeric ZIKV vaccine (ZIKVac), composed of three main portions. At the N’ terminal, there is the ZIKV Envelope protein domain III (EDIII), followed by CD4 + T cell epitopes and finally by overlapping CD8 + T cell epitopes. The vaccine was evaluated with different protein adjuvants at the C’ terminal. Analysis of the biochemical and stability parameters were performed, as well as safety and immunogenicity indicators.

## Results

### ZIKV polyprotein consensus sequence generation

**Figure 1:**
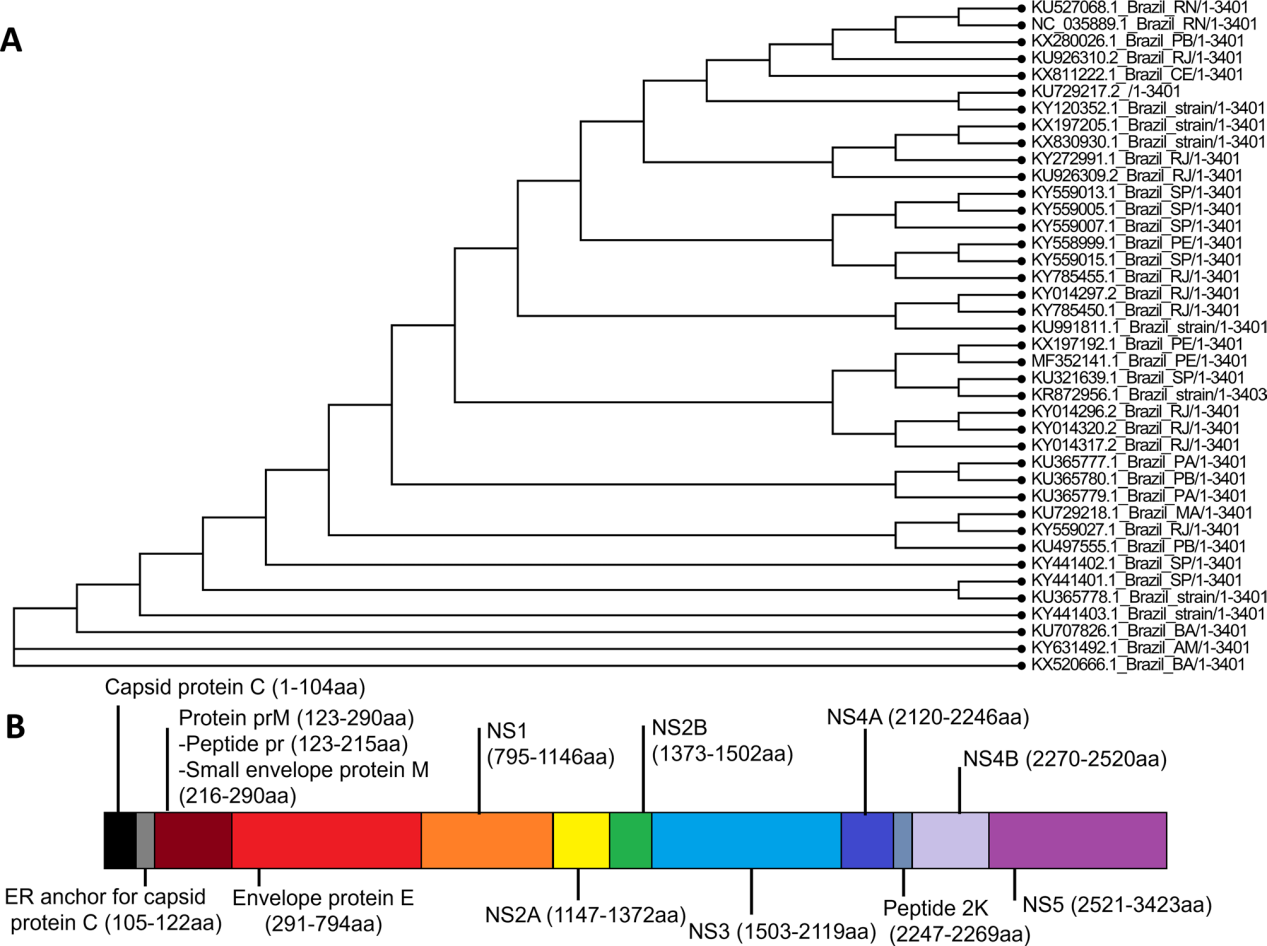
Phylogenetic tree of the 40 complete ZIKV strains used for obtaining the consensus sequence (**A**). (**B**) Polyprotein of the ZIKV from the Brazilian consensus sequence. The ZIKV genome codes for seven non-structural proteins (NS1, NS2A, NS2B, NS3, NS4A, NS4B and NS5) and four structural proteins (Capsid protein C, Protein prM, ER anchor for capsid protein C and Envelope protein E). Protein prM is further digested into Peptide pr and Small envelope protein M. ER anchor for capsid protein C is removed by serine protease NS3. The consensus sequence was obtained from 40 complete Brazilian sequences deposited on NCBI.

Aiming the selection of conserved epitopes, a consensus sequence was generated from 40 complete Brazilian ZIKV sequences available on NCBI (GenBank access number on Supplementary 1). A Phylogenetic tree of all 40 complete sequences was generated, showing high conservancy between them (Fig. 1A). This sequence was later separated into single proteins, which were used as input in FASTA format for the selection of potential type I and II immunogenic epitopes. Figure 1B portraits the obtained ZIKV polyprotein consensus sequence and shows the start and end amino acids of each structural and non-structural protein.

### Selection of CD4 + and CD8 + T cell epitopes

The exploration of multiple epitope prediction servers to identify good CD4 + and CD8 + T cell epitopes may decrease the rate of false positive targets for vaccine construction. Here, at least 3 different platforms based on non-related prediction algorithms were compared. Propred1 and Propred were used to identify the most promiscuous CD8 + and CD4 + T cell epitopes, respectively. We also used the Binding affinity and Immunogenicity tools available on IEDB, and for CD4 epitopes, we also evaluated IFN-γ induction. We first prioritized epitopes that scored above the desired threshold in all servers, however, a considerable part of the selected epitopes was considered hydrophobic and therefore not suitable for synthesis and purification. We then selected only non-hydrophobic peptides, which considerably restricted our options. Hence, it was not possible to select only peptides for all ZIKV proteins with high scores in all servers, so some parameters were prioritized, such as promiscuity. The selected CD8 and CD4 peptides and their HLA coverage, binding affinity score and immunogenicity score are summarized on Tables 1 and 2, respectively.

**Table 1:** Zika virus CD8 peptide selection based on multiparameters using immunoinformatics tools.

Protein	Epitope^0^	Size (aa)	Position	Nº of alleles^1^	% of total alleles^2^	Immunogenicity score (IEDB)^3^	Nº of HLAs w/ Percentile Rank ≤ 20^4^	Nº of HLAs with SMM IC50 ≤ 500 nM^5^	Nº of HLAs with ANN IC50 ≤ 500 nM^6^
Capsid Protein	VSPFGGLKRLP (C_24-34)	11	24–34	17	42.5	0.0036	12	1	1
prM Protein	IFRNPGFALAAAAIA (prM_129-143)	15	129–143	16	34.04	0.3976	49	4	8
Envelope Protein E	SQEGAVHTALA (E_260-270)	11	260–270	10	25	0.23948	15	3	3
Envelope Protein E	VRGAKRMAVLG (E_415-425)	11	415–425	10	25	− 0.21504	15	2	1
Envelope Protein E	MQTLTPVGRLI (E_349-359)	11	349–359	9	22.5	0.13774	26	3	2
NS1	WRSVEGELNAILEENGVQL (NS1_68-86)	19	68–86	11	23.40	0.5434	34	3	3
NS2A	ALIAAFKVR (NS2A_78-86)	9	78–86	5	10.64	0.07299	8	1	1
NS2B	SEVLTAVGLICAL (NS2B_5-17)	13	5–17	22	46.81	0.24318	40	8	7
NS3	VREAIKTRLRTVIL (NS3_209-222)	14	209–222	14	29.79	0.26841	34	6	6
NS4A	GKMGFGMVTLG (NS4A_79-89)	11	79–89	9	22.5	0.01824	24	4	4
NS4A	GAAFGVMEALGT (NS4A_1-12)	12	1–12	12	30	0.17747	29	4	3
NS4B	TPLTLIVAI (NS4B_105-113)	9	105–113	13	27.66	0.20764	10	2	1
NS5	YMWLGARFLEFEAL (NS5_477-490)	14	477–490	17	36.17	0.6005	54	6	6

0—Underlined are the first aminoacids of a 9-mer peptide predicted as having percentile rank ≤ 20 as calculated by IEDB (http://tools.iedb.org/mhci/).

1—Total number of HLA-I covered by the peptide as predicted by Propred1. All 47 alleles were selected, and the percentage of the highest scoring peptides, the Immuneproteasome and the Proteasome filter threshold was set up as 3%.

2—Percentage of HLA-I molecules identified as binders in relation to the total number of HLA-I available.

3—Immunogenicity score obtained with http://tools.iedb.org/immunogenicity/. Positive values are considered immunogenic.

4—Number of HLA that presented percentile rank lower than 20 as predicted by IEDB (http://tools.iedb.org/mhci/).

5—Number of HLAs with SMM IC50 ≤ 500 nM as predicted by IEDB (http://tools.iedb.org/mhci/).

6—Number of HLAs with ANN IC50 ≤ 500 nM as predicted by IEDB (http://tools.iedb.org/mhci/).

**Table 2:** Zika virus CD4 peptide selection based on multiparameters using immunoinformatics tools.

Protein	Epitope	Size (aa)	Position	Nº of alleles^1^	% of total alleles^2^	Immunogenicity (Percentile Rank)^3^	Nº of HLAs w/Percentile Rank < 20^4^	Nº of HLAs w/ SMM align IC50 < 500 nM^5^	Nº of HLAs w/NN align IC50 < 500nM^6^	IFNepitope Score (SVM)^7^
Capsid Protein	HGPIRMVLAILAFLR (C_41-55)	15	41–55	37	72.54	24.3268	21	17	14	0.4521
prM Protein	TTSTWVVYGTCHHKK (prM_71-85)	15	71–85	31	60.8	65.45964	4	0	2	− 0.1269
Envelope Protein E	EYRIMLSVHGSQHSG (E_136-150)	15	136–150	33	64.7	40.45516	13	9	12	− 0.1841
Envelope Protein E	DKLRLKGVSYSLCTA (E_296-310)	15	296–310	34	66.6	46.33096	12	2	12	− 0.3609
NS1	SYFVRAAKTNNSFVV (NS1_121-135)	15	121–135	30	58.8	31.18672	11	12	12	0.3363
NS2A	PFVMALGLTAVRLVD (NS2A_196-210)	15	196–210	29	56.8	42.90484	17	11	14	0.2505
NS2B	FAAGAWYVYVKTGKR (NS2B_116-130)	15	116–130	11	27.50	42.51056	14	5	9	0.5012
NS3	LRGLPVRYMTTAVNV (NS3_236-250)	15	236–250	35	68.6	43.8254	12	8	13	0.4513
NS4A	LGIFFVLMRNKGIGK (NS4A_66-80)	15	66–80	40	78.4	25.47468	21	10	14	0.0979
NS4B	HYMYLIPGLQAAAAR (NS4B_119-133)	15	119–133	18	35.29	42.61168	19	10	14	0.0579
NS4B	VAVSSAILSRTAWGW (NS4B_178-192)	15	178–192	38	74.5	49.2798	13	6	6	0.3106
NS5	LLYFHRRDLRLMANA (NS5_766-780)	15	766–780	36	70.6	38.39304	20	8	12	0.2457

1—Total number of HLA-II covered by the peptide as predicted by Propred MHC-II Binding Peptide Predicition Server. All 51 alleles were selected. The threshold was set up as 1%.

2—Percentage of HLA-II molecules identified as binders in relation to the total number of HLA-II available.

3—Immunogenicity percentile rank obtained with http://tools.iedb.org/CD4episcore/. Lower numbers are considered immunogenic (lower than 66).

4—Number of HLA that presented percentile rank lower than 20 (high affinity).

5—Number of HLA that presented SMM IC50 lower than 500 nM (high affinity).

6—Number of HLA that presented NN IC50 lower than 500 nM (high affinity).

7—IFN-γ induction score. Positive values indicate IFN-γ induction.

ZIKV proteins are poor in CD8 epitopes when compared with CD4 epitopes, as predicted by both Propred1 and the IEDB MHC-I binding prediction server. CD8 epitopes are also inferior when it comes to HLA coverage. Because of that, a threshold of 3% was used for CD8 epitopes on Propred1, while CD4 peptides were selected with a 1% threshold. Moreover, to increase HLA coverage of all ZIKV proteins, more than one peptide was selected for some proteins. NS4A CD8 epitopes GKMGFGMVTLG (NS4A79-89) and GAAFGVMEALGT (NS4A1-12) alone, for example, cover 22.5% and 30% of the available HLA, respectively, but together they cover 52.5%, an average coverage for a CD8 epitope. CD4 epitopes, on the other hand, presented greater HLA coverage and binding affinity in general. Also, 9 out of 12 CD4 peptides were shown to induce IFN-γ production (Table 2).

A total of 13 CD8 + and 12 CD4 + T cell epitopes that covered all structural and non-structural ZIKV proteins were selected for the vaccine construction.

Some of the CD4 + and CD8 + T cell epitopes we selected presented negative scores for immunogenicity (E_415-425) and IFN induction (prM_71-85, E_136-150, E_296-310). However, these epitopes are important for the population coverage of our vaccine, so we performed additional analysis to make sure these epitopes would not bring negative outcomes for the whole vaccine immunogenicity. Therefore, we evaluated the capacity of the mentioned epitopes to induce IL-10 and IL-4, to activate APCs, and to induce proinflammatory response. None of these 4 epitopes were considered inducers of the immunoregulatory cytokines IL-10 and IL-4 and all of them were considered capable of activating APCs. Moreover, all the four epitopes were considered proinflammatory. These results are summarized on Supplementary 3.

### Vaccine construction

**Figure 2:**
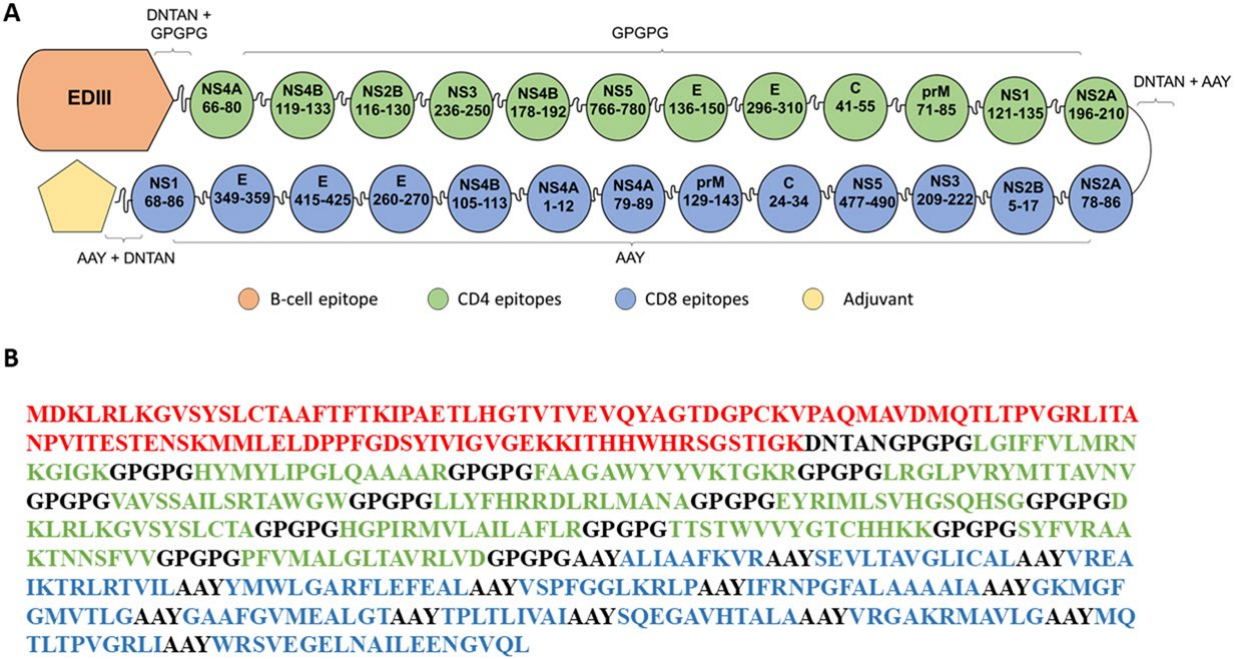
ZIKVac structure showing the position of CD4 (green) and CD8 epitopes (blue) envelope protein E Domain III (EDIII) and adjuvant (yellow pentagon) (**A**). The protein sequence of and ZIKVac sequence without conjugated adjuvants showing the EDIII (red), CD4 epitopes (green), CD8 epitopes (blue) and linkers (black) (**B**). DNTAN linkers were used to separate EDIII and adjuvant from the epitopes, GPGPG linkers were used between CD4 epitopes and AAY linkers between CD8 epitopes.

We rearranged CD4 + and CD8 + T cell epitopes, separated them by proper linkers in order to elect the best vaccine structure based on allergenicity, antigenicity and autoimmunity analyses, secondary and tertiary structure quality and discontinuous epitope scores. EDIII was added to the structure in order to induce B cell response. The DNTAN linker is a loop in protein (LiP) and is appropriate to separate BCR epitopes as there is a need of flexibility between epitopes so they can be recognized directly by B-cells^29^; therefore, we used it to separate EDIII from the rest of the protein. GPGPG and AAY motifs were used to flank CD4 and CD8 epitopes, respectively. We evaluated seven designs (Supplementary 4) regarding their quality of tertiary structure, physical–chemical parameters and antigenicity. We opted to continue the analysis with the structure 6 because it presented the best antigenicity while being predicted to be stable and to have a high expected half-life (Supplementary 5). The EDIII tertiary structure of the final vaccine construct had a high overlap (RMSD 0.845 angstrom) with the EDIII from the crystal structure of ZIKV Envelope protein (PBD: 5JHM), suggesting that the addition of the CD4 + and CD8 + T cell epitopes to the protein structure did not impact the EDIII folding (Supplementary 6). We also added different adjuvants to the vaccine construct to perform our analysis. The final construct (ZIKVac) is shown in Fig. 2 and the ZIKVac sequence associated with adjuvants can be seen in Supplementary 7.

### Evaluation of vaccine autoimmunity induction

Only Flagellin and HBH had sequences with more than six identical amino acids. Flagellin has eight identical amino acids to the human Myosin VI isoform 3, and HBH presents 8 identical amino acids to Histone H1.5. ZIKVac presented sequences with a maximum of only five identical amino acids compared to human proteins (Supplementary 8).

### Allergenicity, antigenicity and physicochemical parameters prediction of ZIKVac

ZIKVac alone and associated with each of the evaluated adjuvants (Flagellin, 50S Rib, HBH and RS09) were considered non-allergenic by AllergenFP server (Table 3). Antigenicity of all structures was evaluated using Vaxijen server, and antigenicity of ZIKVac was predicted to be 0.4749 (Table 3). Interestingly, ZIKVac associated with adjuvants 50S Rib and HBH presented lower antigenicity scores than ZIKVac alone (0.4580 and 0.4427 respectively), while RS09 did not interfere with the vaccine’s antigenicity levels (0.4751). In contrast, flagellin increased ZIKVac’s antigenicity considerably (0.5219). Isoeletric point (pI) ranged between 9.4 and 9.85, which indicates that all constructs are basic in nature. The instability index (II), aliphatic index (AI) and GRAVY were very similar between all structures, meaning that all of them are stable (II < 40), probably thermostable (high AI), and slightly hydrophobic (GRAVY > 0) (Table 3).

**Table 3:** Allergenicity, antigenicity and physicochemical parameters of the ZIKVac and with association with the proteinaceous adjuvants.

Properties	Server	ZIKVac	ZIKVac + flagellin	ZIKVac + HBH	ZIKVac + RS09	ZIKVac + 50S Rib
Length (aa)	ProtParam ExPASy Server	564	847	771	579	702
Probable non-allergen	AllergenFP Server	+	+	+	+	+
Probable antigen Virus, Thr: 0.4	Vaxijen Server	+ (0.4749)	+ (0.5219)	+ (0.4427)	+ (0.4751)	+ (0.4580)
Stable (Instability index)	ProtParam ExPASy Server	+ (25.75)	+ (26.81)	+ (31.20)	+ (26.27)	+ (24.72)
Secondary structure	RaptorX Structure Prediction Server	32%H, 26%E, 40%C	39%H; 22%E; 37%C	41%H; 18%E; 39%C	31%H; 26%E; 41%C	33%H; 24%E; 42%C
Solvent accessibility	RaptorX Structure Prediction Server	41%E, 24%M, 34%B	43%E; 25%M; 30%B	45%E; 22%M; 32%B	42% E; 23%M; 33%B	43%E; 24%M; 32%B
Theoretical pI	ProtParam ExPASy Server	9.82	9.4	9.65	9.77	9.29
Molecular weight	ProtParam ExPASy Server	58.78 kDa	89.06 kDa	81.12 kDa	60.28 kDa	73.02 kDa
Negatively charged residues	ProtParam ExPASy Server	26	60	59	27	52
Positively charged residues	ProtParam ExPASy Server	51	77	87	51	67
Extinction coefficients at 280 nm (if all pairs of Cys residues form cystines)	ProtParam ExPASy Server	78,980 M^−1^ cm^−1^	80,470 M^−1^ cm^−1^	84,940 M^−1^ cm^−1^	80,470 M^−1^ cm^−1^	80,470 M^−1^ cm^−1^
Extinction coefficients at 280 nm (if all pairs of Cys residues are reduced)	ProtParam ExPASy Server	78,730 M^−1^ cm^−1^	80,220 M^−1^ cm^−1^	84,690 M^−1^ cm^−1^	80,220 M^−1^ cm^−1^	80,220 M^−1^ cm^−1^
Estimated half life	ProtParam ExPASy Server	30 h (mammalian reticulocytes, in vitro) > 20 h (yeast, in vivo) > 10 h (Escherichia coli, in vivo)	30 h (mammalian reticulocytes, in vitro) > 20 h (yeast, in vivo) > 10 h (Escherichia coli, in vivo)	30 h (mammalian reticulocytes, in vitro) > 20 h (yeast, in vivo) > 10 h (Escherichia coli, in vivo)	30 h (mammalian reticulocytes, in vitro) > 20 h (yeast, in vivo) > 10 h (Escherichia coli, in vivo)	30 h (mammalian reticulocytes, in vitro) > 20 h (yeast, in vivo) > 10 h (Escherichia coli, in vivo)
Aliphatic index	ProtParam ExPASy Server	89.01	88.02	87.35	88.24	91.15
GRAVY (Grand average of hydropathicity)	ProtParam ExPASy Server	0.233	0.013	0.008	0.215	0.210

### Secondary structure

**Figure 3:**
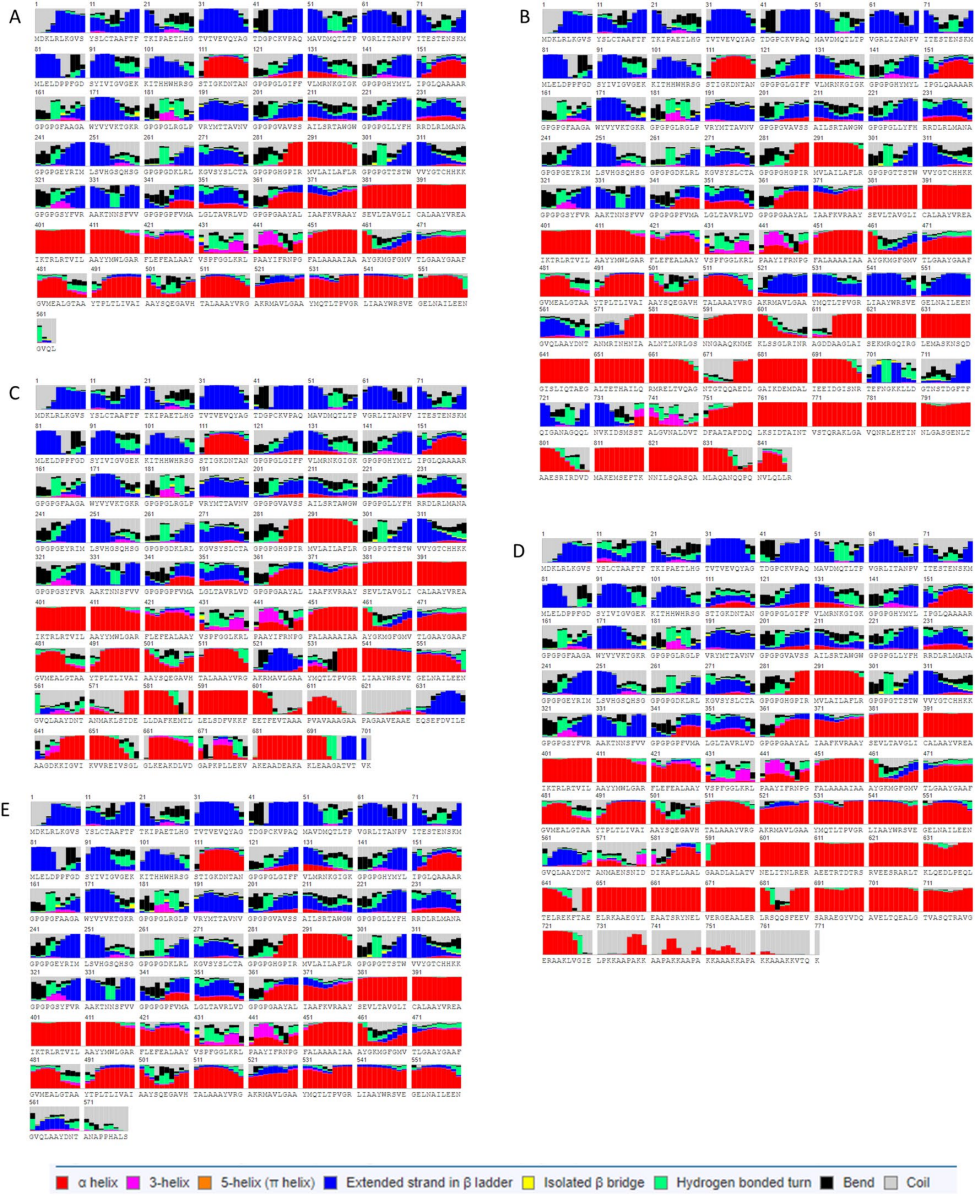
Secondary structure of the vaccine protein alone (**A**) and in association with adjuvants (**B–E**). Helices, coils and beta-sheets are specified in the legend, to note: alpha helix in red, 3-helix in magenta, 5-helix (pi helix) in orange, extended strand in beta ladder in blue, isolated beta bridge in yellow, hydrogen bonded turn in green, bend in black and coil in gray. ZIKVac + Flagellin (**B**), 50S ribosomal (**C**), HBH (**D**), RS09 (**E**).

The secondary structure as predicted by RaptorX reveals that ZIKVac is composed of 32% alpha helices, 26% beta-strands and 40% of coils (Fig. 3). In respect of solvent accessibility, 41% of the protein is exposed, 24% medium and 34% buried. In combination with the adjuvants, the resulting secondary structure varied as shown in Table 5. ZIKVac + flagellin and ZIKVac + HBH contained the highest percentage of alpha helix and the lowest percentage of beta-sheet conformation. The amount of coil formation was about the same in all structures, ranging from 37 to 42%. The addition of the adjuvant did not change the rate of solvent accessibility within the protein.

### Tertiary structure refinement and validation

**Figure 4:**
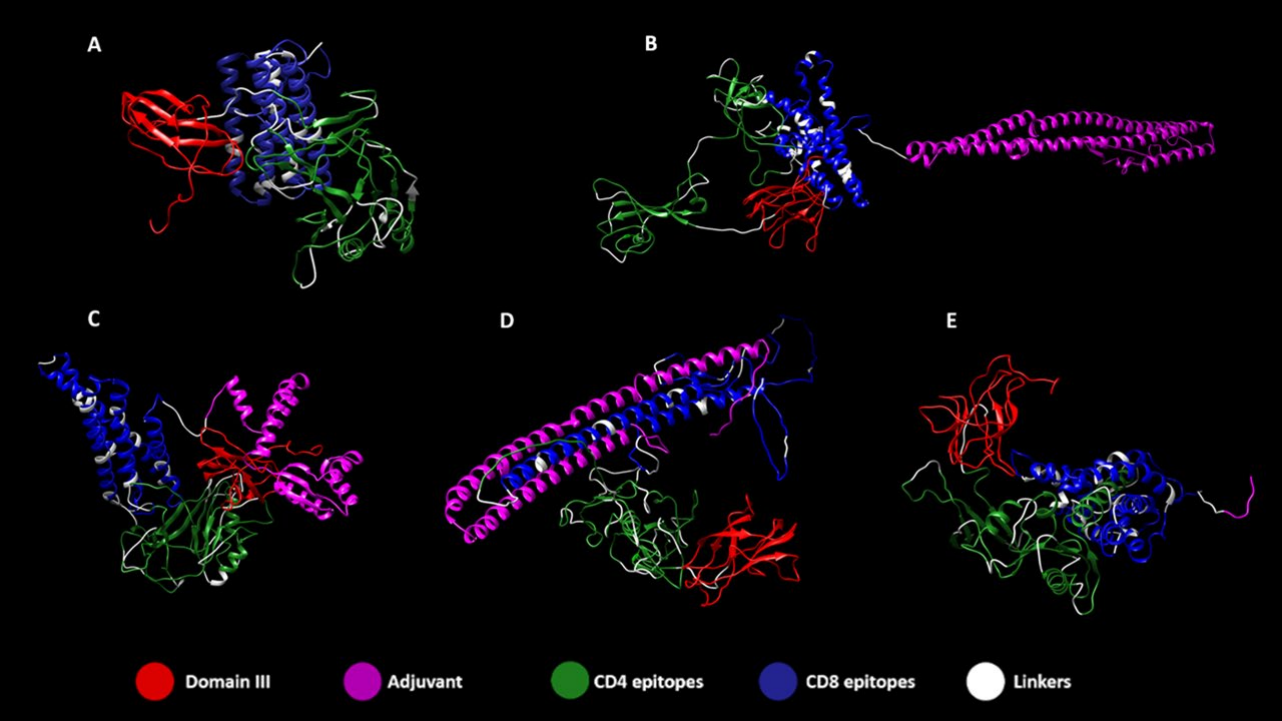
Tertiary structure of ZIKVac (**A**) with highlighted epitopes flanked by linkers and the adjuvants at the C-terminal position, which are the flagellin (**B**), 50S Ribossomal (**C**), Heparin-binding hemagglutinin (HBH) (**D**) and RS09 (**E**). In red is the ZIKV Envelope Domain III, in magenta the adjuvants, in green CD4 epitopes, in blue CD8 epitopes and in white the linkers.

Tertiary structure of proteins was obtained with RaptorX and refined with 3D Refine, followed by validation with RAMPAGE. Figure 4 displays 5 vaccine structures of ZIKVac and ZIKVac associated with the four different adjuvants. Interestingly, in most cases, domain III (Fig. 4A,B,D,E) was separated from the rest of the protein structure, which facilitates antibody recognition and possible attachment with innate cellular receptors, facilitating vaccine intake by the cells. The CD4 and CD8 epitope-rich regions of the protein acquired different conformations according to the adjuvant added. We placed EDIII at the N-terminus to minimize epitope masking by neighbouring domains allowing the greatest number of potential BCRs epitopes to be available. Further, positioning EDIII centrally in the construct may reduce potential effects from neighbouring domains on EDIII folding. The same concept applies for the positioning of the adjuvants at the C-terminus as it can increase the potential TLR binding.

**Figure 5:**
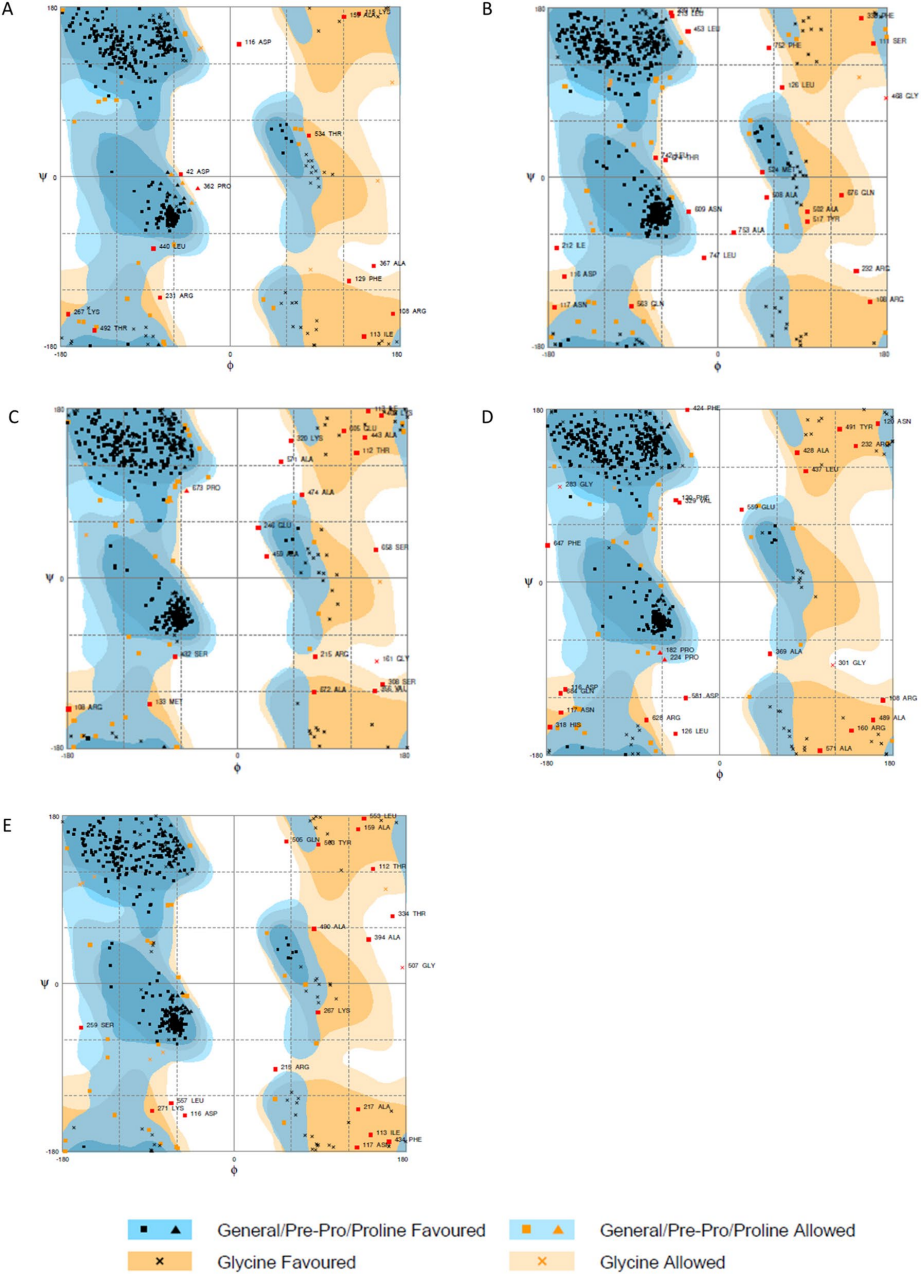
Ramachandran plots of the ZIKVac alone (**A**) and linked with the adjuvants, which are the flagellin (**B**), 50S Ribosomal protein (**C**), Heparin-binding hemagglutinin (HBH) (**D**) and RS09 (**E**). Black squares and triangles in blue areas indicate General/Pre-Pro/Proline Favored, orange squares and triangles in lighter blue areas indicate General/Pre-Pro/Proline Allowed, black Xs in orange regions indicate Glycine Favored and Orange Xs in lighter orange regions indicate Glycine allowed.

Validation of refined tertiary structures showed that all constructs had between 91.0 and 92.7% of residues in favored regions. ZIKVac, ZIKVac + Flagellin and ZIKVac + 50S Rib presented 2.5%, 2.8% and 2.9% of residues in outlier regions, respectively, while HBH and RS09 increased the percentage of residues in outlier regions to 3.6% and 3.3%, respectively (Fig. 5).

### Continuous and discontinuous B-cell epitopes visualization

**Figure 6:**
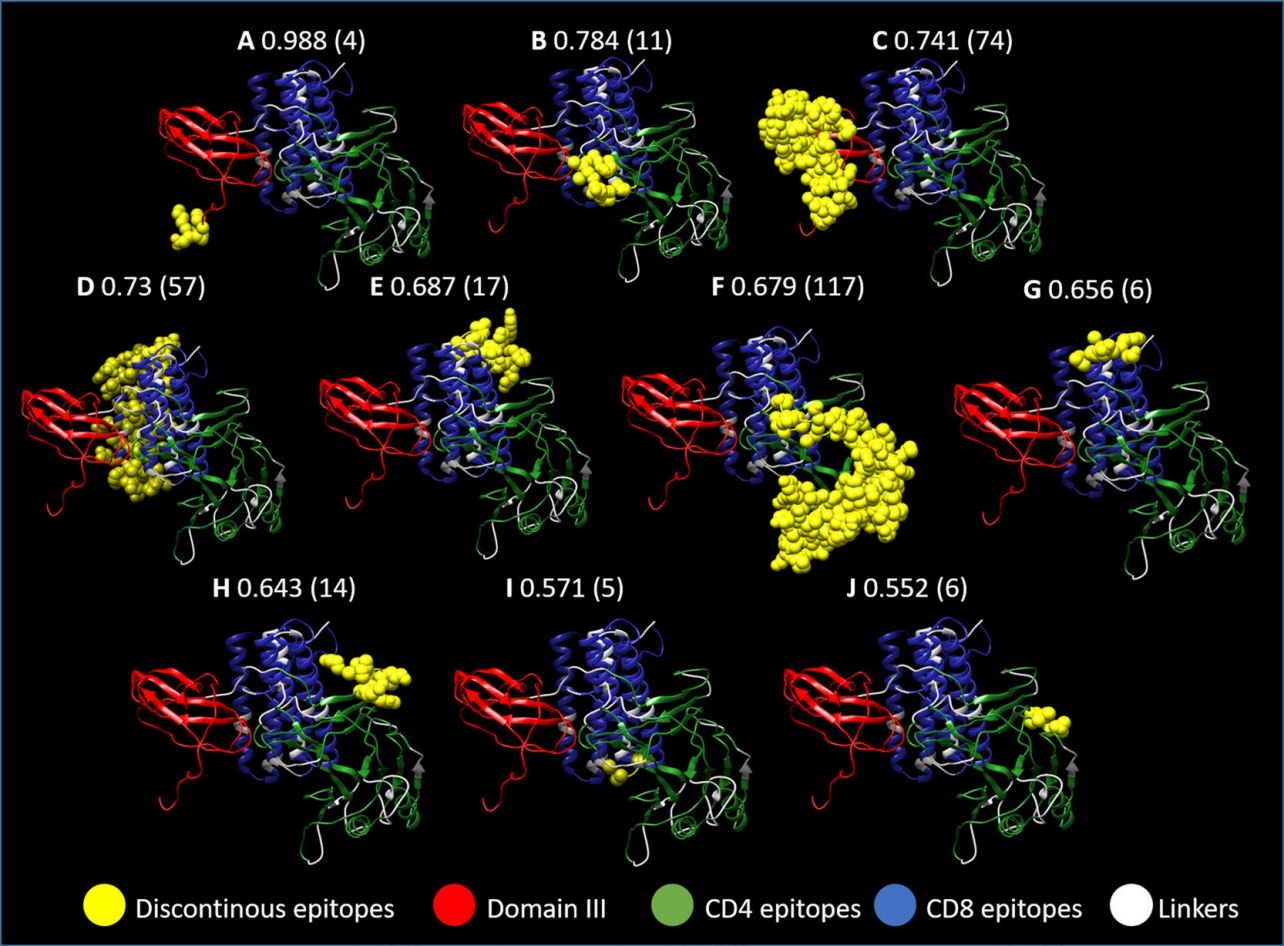
Discontinuous epitopes present in the vaccine construct. Two epitopes are located at the Envelope Domain III portion of the protein (**A** and **C**) with some of the highest scores (0.988 and 0.741) and with 4 and 74 amino acids, respectively. The other discontinuous epitopes are distributed among the CD4 and CD8 epitopes (**B**, **D**–**J**). In yellow are the predicted discontinuous epitopes, in red the domain III from the ZIKV Envelope protein, in green CD4 epitopes, in blue CD8 epitopes and in white the linkers.

Prediction of conformational B cell epitopes from the refined model of ZIKVac were obtained with Ellipro and revealed ten discontinuous epitopes (Fig. 6) and fourteen continuous B cell epitopes (Table 4). The three best continuous and the best and third best discontinuous epitope scores were located at EDIII, corroborating with previous findings that this domain is a good antibody inducer.

**Table 4:** Predicted Linear B cell Epitopes from ZIKVac.

ZIKVac domain	Start–end	Peptide	Number of residues	Score
Domain III	83–94	ELDPPFGDSYIV	12	0.836
Domain III	1–13	MDKLRLKGVSYSL	13	0.777
Domain III	45–69	CKVPAQMAVDMQTLTPVGRLITANP	25	0.774
CD4 epitope domain	154–164	LQAAAARGPGP	11	0.775
Domain III	25–32	AETLHGTV	8	0.739
CD4 epitope domain	200–245	VGPGPGVAVSSAILSRTAWGWGPGPGLLYFHRRDLRLMANAGPGPG	46	0.734
CD4 epitope domain	281–325	GPGPGHGPIRMVLAILAFLRGPGPGTTSTWVVYGTCHHKKGPGPG	45	0.716
CD8 epitope domain	517–564	YVRGAKRMAVLGAAYMQTLTPVGRLIAAYWRSVEGELNAILEENGVQL	48	0.715
Domain III	96–113	GVGEKKITHHWHRSGSTI	18	0.7
CD8 epitope domain	434–450	FGGLKRLPAAYIFRNPG	17	0.687
CD4 epitope domain	134–145	RNKGIGKGPGPG	12	0.658
CD4 epitope domain	253–265	VHGSQHSGGPGPG	13	0.635
CD4 epitope domain	183–187	GPGLR	5	0.567
CD8 epitope domain	471–476	TLGAAY	6	0.564

### Population coverage

In order to confirm whether our vaccine could be effective in different regions and populations, we used the Population Coverage tool available on IEDB online. Only ZIKVac CD4 and CD8 epitopes were considered, not the whole protein construct. It was observed high coverage in several world regions, as indicated on Table 5. Our vaccine’s worldwide population coverage according to the IEDB server was 96.81%, and the highest coverage was in South Asia (98.71%), while the lowest was in South Africa (18.36%). In contrast to South Africa, Central, North, East and West Africa showed 95.44%, 77.17%, 96.18% and 97.95% coverage, respectively. In Central and South America population coverage by our peptides was 92.54% and 94.9%, respectively.

**Table 5:** Population coverage of ZIKVac. The coverage estimation was made using Population Coverage tool available on IEDB online.

Population/Area	Class combined		
	Coverage^a^	Average_hit^b^	pc90^c^
Central Africa	95.44%	50.89	29.93
Central America	92.54%	42.49	26.86
East Africa	96.18%	57.81	32.85
East Asia	89.15%	43.81	23.02
Europe	98.07%	73.1	48.88
North Africa	77.17%	31.37	10.95
North America	95.26%	83.85	52.84
Northeast Asia	95.88%	55.83	31.84
Oceania	97.62%	70.37	43.99
South Africa	18.36%	4.83	3.06
South America	94.9%	79.69	45.58
South Asia	98.71%	57.87	35.16
Southeast Asia	81.05%	33.03	13.19
Southwest Asia	79.38%	32.6	12.12
West Africa	97.95%	74.52	51.06
West Indies	89.23%	44.6	23.21
World	96.81%	68.99	39.46
Average	87.86	53.27	30.82
Standard deviation	18.6	20.31	14.7

^a^Projected population coverage.

^b^Average number of epitope hits / HLA combinations recognized by the population.

^c^Minimum number of epitope hits / HLA combinations recognized by 90% of the population.

### Detection of cytokine-producing CD4 + and CD8 + T cells in response to ZIKV peptides in individuals with previous ZIKV infection

**Figure 7:**
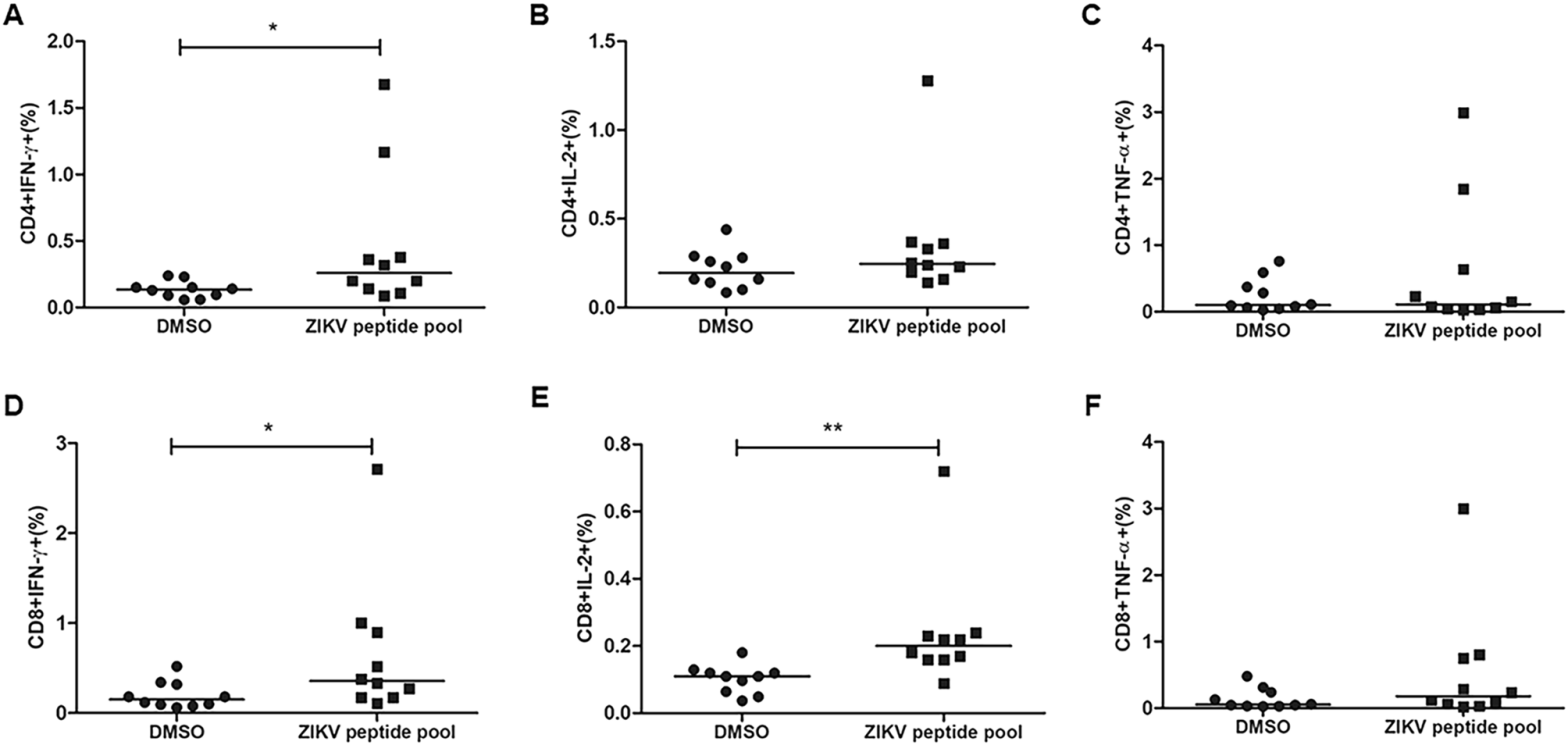
CD4 + and CD8 + T cell responses to ZIKV peptides. PBMC of 10 individuals with previous infection to ZIKV were stimulated for 5 h with the ZIKV peptide pool. After 1 h of stimulation, Brefeldin A was added. The cells were stained with antibodies for the surface markers CD3, CD4 and CD8 and intracellular stained with anti-IFN-γ, IL-2 and anti-TNF-α. IFN-γ, IL-2 and TNF-α production by CD4 + T cells (**A–C**) and CD8 + T cells (**D–F**) was evaluated by flow cytometry. The horizontal lines indicate the medians. Mann–Whitney test was performed to determine statistical significance and *p* < 0.05 was considered significant.

In order to confirm that the predicted peptides could be recognized by T cells from individuals with previous infection with ZIKV and able to induce immune response, we stimulate PBMC of individuals with previous exposure to ZIKV with the pool of 13 CD8 and 12 CD4 peptides selected for the ZIKV vaccine. Cytokine production by CD4 + and CD8 + T cells was assessed after stimulation using flow cytometry (Fig. 7). Gate strategy is represented in Supplementary 9. Both CD4 + and CD8 + T cells showed significant production of IFN-γ (7A, 7D). IL-2 production was also observed by CD8 + T cells (7E), showing a polyfunctionality of CD8 + T cells responses to these epitopes. Some individuals also showed higher TNF-α levels after stimulation, however there was no statistical difference between stimulated and non-stimulated cells for this cytokine (7C, 7F).

## Discussion

ZIKV infection was considered a public health concern in tropical countries, especially in South America due to a rapid growth in the number of microcephaly cases in Brazil caused by the flavivirus in 2015. Although the number of reported cases has waned in the last two years, it could increase anytime with the spread of the mosquito vector during rainy seasons^30^. Considering the potential risks of such infection, it is clear the need of a prophylactic vaccine against it. As the infection is a danger to the fetus, pregnant women in their first trimester would be the main target of such vaccination, as this is the period in which the virus is known to cause more severe neurological damage to the fetus^31,32^. Considering this population, we designed a chimeric multiepitope vaccine that should stimulate the production of specific antibodies, CD4 + and CD8 + T cells to control and prevent viral spread without risk of infection. For selecting the epitopes, we used a consensus sequence derived from all 40 complete Brazilian ZIKV strains available on NCBI by March of 2018. Interestingly, a later analysis showed that this consensus sequence would be the same if all ZIKV genomes obtained in American countries by March of 2018 were considered (about 316 complete and incomplete genomes).

For an effective humoral response, it is fundamental that the vaccine is capable of inducing neutralizing antibodies that lead to inhibition of viral replication, protect against the infection and disease. Flaviviruses structural proteins, especially the Envelope protein, are the main targets for antibody production ^33,34^. The ZIKV EDIII has been used in experimental subunit vaccines, and it was shown to induce neutralizing antibodies against different strains of ZIKV, as well as significant reduction of viral replication when used in combination with alum and Monophosphoryl lipid A (MPL)^35^. Conversely, ZIKV EDIII failed to induce satisfactory inhibition of viral replication in mice when given in the form of DNA, protein or when expressed by a chimpanzee adenovirus^36^, indicating that the choice of adjuvant, for instance, can impact the efficacy of the vaccine. Interestingly, immunization with a specific fragment of EDIII (E298-409) was sufficient to protect BALB/c pups against lethal challenge with ZIKV strains R103451 and FLR^37^.

With that in mind, we chose ZIKV EDIII to cover our vaccine’s B cell response. EDIII from Dengue virus and other flaviviruses have been previously reported to induce neutralizing antibodies^38–40^. Similarly, ZIKV EDIII specific antibodies have also shown neutralizing activity, while domains I and II have presented either modest or none^11,41,42^. EDIII was therefore a promising choice for a vaccine construct. The inclusion of the ZIKV EDIII to the multiepitope vaccine structure is a novelty of our work.

Moreover, a chimeric protein-peptide structure may be more efficient in eliciting neutralizing antibodies than the peptides alone, due to the fact that it is more probable to find discontinuous epitopes in a protein structure, while peptides usually contain only linear epitopes. Our vaccine structure revealed ten discontinuous epitopes and fourteen continuous epitopes. Interestingly, the two discontinuous epitopes (1st and 3rd best discontinuous epitopes) with the best scores were located at EDIII.

Even though the main purpose of EDIII in our vaccine is to induce neutralizing antibodies, it can also be degraded by different types of proteasomes and/or by cellular enzymes and be presented to T cells after binding to proper MHC^43^. Salvador et al. (2019) found by IEDB NetMHCpan method epitopes for CD8 + (MMLELDPPF) and CD4 + T cells (QMAVDMQTLTPVGRL) that are present in the EDIII of our vaccine^44^. By utilizing different methods (NetCTL1.2 and Ellipro Protrusion index), Mirza and colleagues found 4 epitopes present in EDIII, of which 3 are CD8 epitopes (CTAAFTFTK, GTVTVEVQY, STENSKMML), and 1 is a linear B cell epitope (LKGVSYSLCTAAFTFTKI)^45^. Furthermore, Cunha-Neto and collaborators explored a new mathematical model of structural entropy and found that KMMLELDPPF is a CD8 epitope, and interestingly this epitope is also part of EDIII^46^. Several epitopes derived from EDIII were found in previous reports to induce CD8 + and CD4 + T cell responses, demonstrating that the protective potentialities of ZIKVac can extrapolate our predictions^45–47^.

Regarding the cellular immune response of ZIKVac, we included 12 HLA class II peptides derived from all structural and non-structural proteins of ZIKV. CD4 + T cell differentiation into effector memory and terminally differentiated cells was observed in patients in the acute phase of ZIKV infection ^48^, which suggests that a CD4 + T cell epitope-based vaccine may contribute to eliciting a cytokine inducing response. Apart from binding affinity and promiscuity, we also considered IFN-γ induction capability in order to select our vaccine’s CD4 + T cell peptides, since high levels of this cytokine during the acute phase of ZIKV infection appears to be related to viral clearance and mild disease^49^. Moreover, some of the selected CD4 + T cell epitopes were described previously as being also CD8 and B cell epitopes, and interestingly, AAGAWYVYV derived from NS2B, induced IFN-γ as detected by IFN-γ ELISPOT^50^.

CD8 + T cell epitopes are critical components of our vaccine construct, since most studies support the idea that these cells help control infection through viral clearance. In mice with IFN receptor-competent T cells and dendritic cells, CD8 + T cells demonstrated a protective role during ZIKV infection^50^. CD4 + and CD8 + T cells proliferation assessment in mice infected with ZIKV induced a strong adaptive immune response, which helped prevent infection in the brain and testes. Moreover, in ZIKV-infected pregnant mice, CD8 + T cell responses were decreased in comparison with non-pregnant mice, which suggests that the cell-mediated immunity during pregnancy may contribute to preventing virus spread to the fetus^51^. In accordance with our study, Cunha-Neto et al. (2017) described the epitope QEGAVHTAL (from E protein) as potentially capable of inducing CD8 + T cell response by using structural entropy-based predictions^46^. Furthermore, some epitopes placed in the CD8 portion of ZIKVac (SPFGGLKRLPA, MAVDMQTLTPV) were found to induce IFN-γ response in vitro^47,50^. Due to the limited amount of CD8 epitopes and aiming to increase population coverage as much as possible, we opted to use overlapping CD8 peptides as it would make possible to include a higher number of epitopes in a shorter amino acid sequence; however, this resulted in some peptides longer than 11 amino acids. In these cases, we would expect that intracellular peptidases, such as Endoplasmic reticulum aminopeptidase 1 and 2 (ERAP1 and 2) would play a role in cleaving these epitopes in order to allow their coupling with the HLA type I binding groove^43^. Some of the epitopes we selected showed negative scores for some of the parameters we analyzed. However, we believe they will not bring negative outcomes to the vaccine immunogenicity, since none of them were considered inducers of the immunoregulatory cytokines IL-10 and IL-4. Prediction results showed these epitopes are also capable of activating APCs, which can be a booster for activation of innate immunity by our vaccine. Moreover, all the four epitopes were considered proinflammatory. This can indicate that even if they are not able to induce IFN, they can be inducers of other proinflammatory cytokines such as TNF-α.

Other in silico vaccine structures based on epitope prediction have been designed since the association of ZIKV with severe disease and fetus malformation^52–56^. Differently from our vaccine structure, some of them were not based on consensus sequences^54,56^. Alam et al., (2016) and Franco et al., (2017) developed vaccines based only on the Envelope protein and NS5 protein, respectively, and Pandey et al., (2018) focused on E, C prM and NS1 proteins^52,53,54^. Similar to our approach, in which we included at least one peptide from each ZIKV structural and non-structural protein, Prasasty et al., (2019) and Shahid et al., (2020) explored almost all the ZIKV proteins^55,56^. Prasasty et al., (2019) did not have the whole vaccine construct analyzed for parameters such as autoimmunity, allergenicity and stability, but only the peptides alone^55^. Our analysis showed that the whole protein assessment is important to determine the assembly viability, since addition, deletion or changes in the order of peptides disposition can affect vaccine’s efficacy and safety. Finally, our vaccine assembly is the only one that includes a protein domain, which we believe is an important differential feature and can be advantageous when it comes to the presence of discontinuous B cell epitopes and induction of a more robust and specific antibody response.

Interestingly, some of our epitopes were also found by Pandey et al., (2018) and Prasasty et al., (2019), even though they used different methods than ours^54,55^. One of our CD4 + T cell epitopes (HGPIRMVLAILAFLR) and two of our CD8 + T cell epitopes (IFRNPGFALAAAAIA; VRGAKRMAVLG) are contained in CD4 + T cell epitopes selected by Pandey et al., (2018) (LGHGPIRMVLAILAFLRFTA; VENWIFRNPGFALAAAAIAWLLGSS; VRGAKRMAVLGDTAWD). Similarly, three CD8 + T cell (MVLAILAFL; YMWLGARFL; FAAGAWYVY) and two CD4 + T cell epitopes (YRIMLSVHG; LYFHRRDLR) selected by Prasasty et al., (2019) are contained in five of our CD4 epitopes (HGPIRMVLAILAFLR; YMWLGARFLEFEAL; FAAGAWYVYVKTGKR; EYRIMLSVHGSQHSG; LLYFHRRDLRLMANA). The epitope YRIMLSVHG selected was also selected by Alam et al., (2016)^52^.

Adjuvant use in vaccine constructs is important for immune response enhancement. We tested our vaccine with 4 different adjuvants in order to find one that could increase its antigenicity. We selected 4 TLR agonists as adjuvants because activation of these receptors leads to innate cell stimulation, cytokine production, receptor expression, dendritic cell maturation and subsequently T cell priming^57^. These proteins were chosen based on other studies that showed that they presented promising results as adjuvants. For instance, flagellin is a TLR5 agonist derived from *Bacillus subtilis* subsp. *spizizenii* strain W23 (GenBank access number: ADM39502.1) retrieved from NCBI. It has been previously shown that flagellin induced robust humoral response and pro-inflammatory cytokine production when administered with a whole inactivated influenza vaccine in mice^58^. Heparin-binding hemagglutinin (HBH) (UniProtKB—P9WIP9) is a TLR4 agonist derived from *Mycobacterium tuberculosis* H37Rv, while 50S ribosomal protein L7/L12 (UniProtKB—P9WHE3) is also a molecule derived from *Mycobacterium tuberculosis* H37Rv and is described as a TLR4 binder. When used as adjuvant for a therapeutic cancer vaccine, HBH favored Th1 polarization and dendritic cell (DC) function^59^. 50S ribosomal protein L7/L12 also enhanced Th1 polarization in a DC-based tumor immunotherapy^60^. Finally, RS09 (APPHALS) is a short synthetic LPS peptide mimics designed using Phage Display Libraries and described as capable of binding to TLR4^61^. RS09 was previously used as adjuvant in an HIV-1 vaccine that showed promising results in mice^62^. Our autoimmunity analysis showed that the only portion of the vaccine that had more linear amino acids similarity with human proteins was present on the Flagellin adjuvant portion (MAKEMSEF). However, this peptide is not long enough to induce a CD4 type response and is not predicted to bind to any of the HLA class I molecules considered in this study (the sequence is too short to be assessed in respect to its CD4-inducing properties). Without the help of T-cells, it is not likely that this peptide will induce a B cell response^63^. Furthermore, flagellin has been used as adjuvant in vaccines against bacterial pathogens^64,65^, parasitic organisms^66,67^, cancer^68^ and virus infection, such as influenza^69^, WNV^70^, DENV^71^ and HIV^72^. This adjuvant was also the one that increased vaccine antigenicity score the most in relation to the other 3, indicating that it is a potent adjuvant, however safety experimental evaluation is necessary.

Even though we presented our ZIKVac in combination with 4 protein TLR agonists, its potential application is not limited to them. ZIKVac could also be expressed or synthesized without the adjuvant domain and be administered with other types of adjuvants, such as MPL. MPL is a TLR4 agonist dependent on TRIF signaling that has been used for influenza vaccination in Brazil due to its not so aggressive immunogenicity in comparison to LPS and for reducing the dose of the antigen in vaccination regimens^73^. ZIKVac could also be used with aluminum, which is the most frequent adjuvant in licensed vaccines, showing a great safety record^74,75^. The combination of both adjuvants can lead to a more balanced Th1/Th2 response and resulted as the best adjuvant scheme when compared to either adjuvant alone or MF59^35^. Thus, we initially plan to express the proposed vaccine in eukaryotic cells and test it as a protein adjuvanted with alum and MPL in mice. However, it would also be particularly interesting to test it as a nanoparticle encapsulated mRNA or carried by a Hepatitis B core Antigen (HBcAg)-VLP since both strategies have been used for ZIKV vaccines and showed promising data^12,76^.

An ideal vaccine should have global coverage. In South America, where ZIKV infection has been more prevalent^77^, our vaccine’s peptides covered 94.9% of the population. The highest coverage was in South Asia (98.71%), and the lowest coverage was found in South Africa (18.36%). However, the coverage in North and Central Africa were 77.17% and 95,44%, respectively. In general, world coverage was 96.81%, indicating that these epitopes are promiscuous and might be recognized by various populations worldwide. This is important because the virus may spread to unexpected regions, and a universal vaccine would be crucial to stop any novel epidemics. Similarly, when we evaluated HLA coverage of our peptides using Propred1 and Propred server, we found that ZIKVac’s CD4 peptides covered 100% of the HLA molecules available in the server and CD8 epitopes covered 46 of the 47 available molecules. HLA-A*1101 (predominantly found among the Asian population) was the only molecule to which none of our peptides were predicted to bind. Experimental validation of predicted CD8 + T cell DENV and hepatitis B virus (HBV) epitopes for this molecule showed that the predicted and laboratory results did not match^78^. This could be explained by the fact that HLA molecules frequently observed among the Caucasian population (e.g. HLA-A*0201) have a larger amount of data available than Asian predominant HLA molecules, and the efficacy of epitope prediction algorithms depend directly on the training data available for each HLA molecule^78^.

Despite the limitations of prediction servers, various studies have been successful with experimental validation of predicted epitopes. Iwai et al., (2003) tested synthetic peptides of Paracoccidioidomycosis (PCM) gp43 protein selected using the TEPITOPE algorithm and found that peptide recognition by PCM patients correlated with the promiscuity of HLA-DR binding prediction^79^. Similarly, Farrell et al., (2016) found that predicted promiscuous *Mycobacterium bovis* epitopes significantly induced IFN-γ secretion from infected cattle T cells when compared to epitopes with low promiscuity^80^. Moreover, Ribeiro et al., (2010) developed a DNA vaccine which encodes 18 HIV subtype B CD4 + T-cell epitopes^81^. The DNA vaccine induced robust CD4 + and CD8 + T cell responses in mice expressing different class II HLA alleles, and 16 of the 18 coded epitopes were recognized by PBMC from HIV infected patients. These studies suggest that in silico prediction approaches can guide the selection of conserved and antigenic epitopes that can be recognized by the target population, thereby reducing the time to develop effective vaccines. In order to test whether our peptides are capable of inducing immune response, we performed a preliminary in vitro assay and evaluated the production of cytokines after stimulation of PBMCs of individuals with previous ZIKV infection with a pool of the 25 ZIKV peptides. Both CD4 + and CD8 + T cells showed significant production of IFN-γ upon stimulation, confirming what was predicted using the IFN epitope prediction server. IL-2 production was also detected by CD8 + T cells. The results indicate that the ZIKV peptides of our in silico designed vaccine were presented to T cells in the course of natural ZIKV infection and generated memory responses that could be boosted by peptide stimulation in vitro leading to cytokine production by CD4 + and CD8 + T cells. These data support our epitope prediction strategy and reinforce the vaccinal potential of these peptides.

## Conclusions

ZIKV infection is especially concerning in pregnant women, and more elaborated approaches are needed in order to attend this population. We designed a chimeric multidomain Zika vaccine intending to cover all the protective immune responses against ZIKV, targeting specific CD4 + , CD8 + T cells and B cells. This in silico approach gave us many promising hits. In vitro tests showed that our ZIKV peptides can be recognized by T cells and induce immune response. We intend on later synthesizing the whole vaccine structure to validate its potential in vivo as well.

## Material and methods

### Brazilian strains consensus sequence

All complete Brazilian ZIKV strains (by March of 2018) were obtained from NCBI (https://www.ncbi.nlm.nih.gov/) and downloaded in FASTA protein format. The sequences access numbers are listed on Supplementary 1. The alignment was done by the software ClustalX2.1, and the consensus sequence was obtained with Jalview (version 2.11). This sequence was later separated into single proteins, which were used as input in FASTA format for the selection of potential HLA type I and II immunogenic epitopes. After aligning the sequences with ClustalX, we built a phylogenetic tree using FigTree v1.4.4.

### HLA class I-restricted epitope prediction

In order to find the best potential ZIKV type I epitopes for immunization purposes, three binding peptides prediction platforms were compared: Proped1 (http://crdd.osdd.net/raghava/propred1/), IEDB Class I Immunogenicity (http://tools.iedb.org/immunogenicity/) and IEDB MHC-I Binding Predictions (http://tools.iedb.org/mhci/), following this order. Epitopes that were found promising in all 3 platforms were considered for further investigation. Propred1 highlights antigenic epitopes in a given protein sequence in 47 MHC alleles including HLA and H2 alleles^82^. All available alleles were selected for maximum population coverage, and the threshold of 3% was selected for the immunoproteasome and the proteasome filters. IEDB MHC-I Binding Predictions combines information from several binding prediction algorithms. The IEDB recommended method was used as it includes the Artificial Neural Network (ANN), Stabilized Matrix Method (SMM) and Combinatorial Library methods. The IEDB server has a list of the 27 most frequent class I alleles available (97% population coverage)^83^, which were used for the prediction considering 9mer peptides. ANN and SMM indicate the predicted binding affinity between HLA class I and a given epitope in nM, so low values (< 500 nM) of ANN and SMM indicate that the peptide and the HLA molecule may have a stable interaction. Finally, to measure peptide immunogenicity, IEDB Class I Immunogenicity resource was used. Peptides with immunogenicity score (> 0.15) were considered as immunogenic. In this tool, the amino acids properties as well as their position inside a given peptide are used to calculate the immunogenicity score of a given epitope^84^.

### HLA class II-restricted epitope prediction

In order to select the best potential ZIKV CD4 + T epitopes, the binding affinity to HLA class II molecules, promiscuity, immunogenicity and predicted IFN-γ induction were evaluated. Epitopes found promising in all four parameters were selected for further analysis. Initially, CD4 + T cell epitopes for ZIKV structural and non-structural proteins were predicted using Propred MHC-II Binding Peptide Prediction Server (http://crdd.osdd.net/raghava/propred/). 15-mer length epitopes were predicted for all 51 HLA class II alleles available in the server with 1% threshold. Propred server is based on matrices for the alleles obtained from a pocket profile database previously described^85^. Epitopes were selected considering the maximum number of alleles that bind to the epitope. The IEDB server (http://tools.iedb.org/mhcii/) was then used to determine epitope affinity to HLA class II alleles based on percentile rank and IC50 values. Percentile rank ≤ 20 was considered for selecting promiscuous peptides and IC50 ≤ 500 nM was considered for peptides with intermediate to high binding affinity. Epitope immunogenicity was evaluated using the IEDB server (http://tools.iedb.org/CD4episcore/), considering percentile rank lower than 66 for immunogenic peptides^86^. Finally, epitope potential to induce IFN-γ was evaluated using IFNepitope server (http://crdd.osdd.net/raghava/ifnepitope/), and peptides with positive values were considered to be capable of inducing IFN-γ production.

### Prediction of induction of IL-10, IL-4, proinflammatory response and APC activation

The capacity to induce IL-10 and IL-4, activate APCs, and induce proinflammatory response was evaluated for epitopes that presented negative immunogenicity scores in order to check if they would bring a negative outcome to the overall vaccine immunogenicity. Prediction of induction of IL-10 and IL-4 were performed with the downloaded IL10pred platform^87^ and with the IL-4Pred online server (https://webs.iiitd.edu.in/raghava/il4pred/index.php)^88^, respectively. The capacity to activate APCs and induce proinflammatory response was performed with the VaxinPAD online server (https://webs.iiitd.edu.in/raghava/vaxinpad/index.php)^89^ and ProInflam online server (http://metabiosys.iiserb.ac.in/proinflam/index.html)^90^, respectively. A 0.5 threshold was used for all these parameters as previously described^91^.

### Vaccine construction

The selected CD4 + and CD8 + T cell epitopes were chosen from a set of peptides predicted by the aforementioned servers considering population coverage, immunogenicity score and MHC binding affinity. ZIKV Envelope E Domain III (EDIII) (consensus) was added to the structure as well. The epitopes and EDIII were flanked by linkers, short amino acid sequences, which have been described to be cleaved by proteasomes and lysosomes so the epitopes can bind to HLA class I and HLA class II, respectively^92^. In order to find a sequence of epitopes that could achieve the highest antigenicity while maintaining protein stability, we evaluated the best order in which the epitopes could be organized before choosing the final construct, such as EDIII-CD4-CD8, EDIII-CD8-CD4 and CD8-CD4-EDIII.

Aiming to increase vaccine antigenicity, its structure was evaluated with different combination of protein adjuvants, such as flagellin, Heparin-binding hemagglutinin (HBH), 50S ribosomal protein L7/L12 (50S rib) and RS09, a synthetic LPS peptide mimics designed with Phage Display Libraries^61^. Antigenicity, stability, safety and some physicochemical parameters of all of them were predicted using different platforms described in the following methods.

To separate the epitopes, protein domain and adjuvant, proper linkers were used. DNTAN linkers were used to separate the adjuvant from the vaccine epitopes, and/or the EDIII from the other parts of the vaccine^93^ while GPGPG linker was used to separate CD4 epitopes^94^, and AAY was used to individualize CD8 epitopes^92,95^. We chose GPGPG linkers considering it induced broad MHC class II-restricted T cell responses in mice vaccinated with an epitope-based HIV vaccine previously designed by our group^81^. The DNTAN linker was chosen to give flexibility to the different parts of the vaccine construct because it is considered a loop in protein (LiP)^96^, which is important to mediate biological processes such as signaling and protein–ligand binding^97^.

### Population coverage

Assessment of vaccine epitopes coverage is important to predict which population or populations are going to benefit from the selected epitopes. To this end, we used the IEDB online server Population Coverage (http://tools.iedb.org/population/) developed by Bui et al., (2006)^98^, utilizing the Allele Frequencies Net Database that contains a study of 1081 populations and 1066 alleles of the HLA gene^99–101^. The HLA class I and class II restricted epitopes category was selected, and the 12 CD4 + and 13 CD8 + T cell epitopes were included. All world regions were selected to assess all possible areas that could be covered by the vaccine.

### Vaccine safety

If not evaluated properly, vaccines can become allergens and/or induce autoimmunity. In order to assess if the proposed vaccine could be a potential allergen, the protein FASTA sequences were used as input in the allergenicity prediction server Allergen FP v.10 (http://ddg-pharmfac.net/AllergenFP/index.html). Allergen FP v.1.0 method is based on four steps algorithm considering the physicochemical properties of individual amino acids in a given protein. It applies a universal fingerprint approach that is alignment-free and has shown accuracy of 88%^102^.

Additionally, if self-antigens are found in the vaccine sequence, the humoral and cellular responses induced against the vaccine may target self-proteins, leading to autoimmunity. In order to increase the chances that ZIKVac would not present such complication, blastp was performed with the NCBI platform (https://blast.ncbi.nlm.nih.gov/Blast.cgi?PAGE=Proteins), using the parameters described elsewhere^103^. Identical continuous human sequences smaller than 6 amino acids were discarded as the likelihood of such pairing is high, but not significant^103^.

### Antigenicity

Antigenicity of the final vaccine construct was predicted using VaxiJen v2.0 (http://www.ddg-pharmfac.net/vaxijen/VaxiJen/VaxiJen.html), with a threshold of 0.4 and “virus” as target organism or with ANTIGENpro (http://scratch.proteomics.ics.uci.edu).

### Physicochemical properties and solubility

Evaluation of physicochemical characteristics of proteins are crucial for proper protein expression, separation, purification, storage and additional analyses. Thus, the vaccine's physicochemical properties were analyzed with ProtParam ExPASy Server^104^, https://web.expasy.org/protparam/. With the ProtParam tool, it was observed the protein molecular weight, theoretical pI, extinction coefficients, estimated half-life, instability index, aliphatic index and GRAVY (Grand average of hydropathicity).

### Secondary and tertiary structure

Tertiary and secondary structures of all constructs were predicted using RaptorX Structure Prediction (http://raptorx.uchicago.edu/StructurePrediction/predict/). This server was chosen because it is capable of predicting tertiary structures of proteins that have less than 30% similarity with proteins deposited in PDB (Protein Data Bank), i.e. without any similar homologs; additionally, it also provides information related to the protein secondary structure, solvent accessibility and a quality score, which can be used to select the best model. RaptorX performance in CASP9 (Critical Assessment of protein Structure Prediction 9) was superior than the other participating servers when complex targets were evaluated^105^.

### Tertiary structure refinement and validation

Tertiary structure refinement was conducted with 3D Refine web server (http://sysbio.rnet.missouri.edu/3Drefine/). The PDB file obtained with RaptorX was used as input. After complete running, the server provides 5 refined models with values of 3D refine score, GDT-TS GDT-HA, RMSD (root-mean-square-deviation), MolProbity, RWPlus. The 3D refine and RWplus scores are the model potential energy after refinement; GDT-TS and GDT-HA indicate how conservative was the refinement; RMSD indicates in angstroms the deviation score of the model; MolProbity indicates how physically realistic the refined model is.

3D Refine algorithm combines 2 steps of minimization that includes optimizing Hydrogen Bonding Network and Energy Minimization. The algorithm refinement capacity at local and global measurement of structural quality has been shown in CASP8, CASP9 and CASP10 with superior results compared to other refinement methods^106^.

After refinement, protein structure was validated by RAMPAGE (http://mordred.bioc.cam.ac.uk/~rapper/rampage.php), which provides the Ramachandran plot. The Ramachandran plot will present if the Ѱ and Φ angle clusters are well positioned and points out if there is any abnormality resulted from the protein refinement. The torsion angles Ѱ and Φ characterizes the spatial conformations of each amino acid residue, and the Ramachandran plot groups information about the number of residues in favored region, allowed region or outlier region^107^.

Based on the refinement scores and Ramachandran favored and disfavored regions, the best models were chosen for representation and discontinuous epitope prediction. The selected models were edited using Chimera v1.13.1.

### Continuous and discontinuous B cell epitopes

Elucidation of conformational vaccine epitopes are important as BCR recognizes epitopes without presentation or previous processing. In addition, protein folding may hide linear B-cell epitopes. Tertiary structure of the protein was used as input in the ElliPro server (http://tools.iedb.org/ellipro/), and minimum score was set as 0.5 (default) and maximum distance was 6 Angstrom (default). ElliPro shows as output potential sequences of discontinuous epitopes as well as their location in the tertiary protein structure. This prediction is based on Thornton’s method^108^ and also includes residue-clustering algorithm. It is taken into consideration that, in case the protein is considered an ellipsoid, sequences that protrude from the ellipsoid are more freely recognized by antibodies^109^. ElliPro has the best AUC, 0.732, when compared with six other structure-based methods after testing of discontinuous protein epitopes from a benchmark dataset^110^.

### ZIKV peptide synthesis

All 13 CD8 and 12 CD4 ZIKV peptides predicted in this work were synthesized through solid phase chromatography using 9-fluorophenylmethoxy-carbonyl strategy, with the C-terminal carboxyl group in amide form. Peptide purity and quality were done by reverse-phase high performance liquid chromatography by Biointech/Pepmic Co. (Brasília, Brazil/China), considering the peptides with purity > 95%. Peptides were dissolved in dimethylsulfoxide (DMSO). All 25 peptides were pooled together and after testing different concentrations, 25 µg/mL was determined as the best concentration for usage.

### Human blood samples collection and ZIKV infection detection

In order to validate the immunogenicity of the selected peptides, blood samples from 10 convalescent donors previously infected with ZIKV were used to carry out in vitro assays (Supplementary 2). ZIKV infection was confirmed by plasma and/or urine detection of ZIKV RNA by RT-qPCR during the acute phase of infection. Blood collection was conducted between two and three years after the acute phase of infection diagnosis at Federal University of Goiás (Goiânia, Brazil). IgM and IgG serology for ZIKV was performed after blood collection using the double-path TR DPP® Zika IgM/IgG immunochromatographic test at the Bio-Manguinhos Recombinant Technology Laboratory in Osvaldo Cruz Foundation (Rio de Janeiro, Brazil). Peripheral Blood Mononuclear cells (PBMCs) were isolated by standard density gradient centrifugation on Ficoll-Paque™ Plus (GE Healthcare) and cryopreserved in a 90% fetal bovine serum (FBS) (GIBCO, Grand Island, USA) and 10% DMSO (Sigma-Aldrich, St Louis, USA) solution. Cells were stored in liquid nitrogen until usage.

### PBMC stimulation with ZIKV peptide pool, surface phenotype and intracellular cytokine staining

PBMCs from patients previously infected with ZIKV were thawed and incubated at 37 °C with 5% CO_2_ for 2 h. Cells were then washed and distributed at a concentration of 2 × 10^6^ cells/mL in polypropylene tubes with RPMI 1640 medium (Lonza, Walkersville, USA) supplemented with 0.1% penicillin, 0.1% streptomycin (GIBCO, Grand Island, USA) and 5% FBS. Cells were stimulated for 5 h with 25 µg/mL of the ZIKV peptide pool containing all 25 CD4 and CD8 peptides at 37 °C with 5% CO_2_. After 1 h of stimulation, 1 µg/mL of Brefeldin A was added (BD Biosciences). DMSO was added to non-stimulated cells as negative control and Staphylococcal enterotoxin B (SEB) was used as positive control. Cells were washed with PBS and then with FACS buffer and stained with surface antibodies (CD3-APC-Cy7 (clone SK7, BD Pharmingen); CD4-PerCP-Cy5.5 (clone RPA-T4, BD Pharmingen); CD8-PE (clone HIT8a, BD Pharmingen). Cells were incubated at 4 °C for 30 min and then washed twice in FACS buffer. 100 µl of Cytofix/Cytoperm (BD Biosciences, San Diego, USA) was added and cells were incubated at 4 °C for 20 min. Cells were then washed with Perm/Wash 1X (BD Biosciences, San Diego, USA) and stained with intracellular cytokine antibodies (IFN-γ-FITC (clone B27, BD Pharmingen); IL-2-APC (clone 5344.111, BD FastImmune); TNF-α-PE-Cy7 (clone MAb11, BD Pharmingen) at 4ºC for 30 min. Cells were washed twice with Perm/Wash 1X and fixed in 2% paraformaldehyde solution (Sigma-Aldrich, St. Louis, USA). Acquisition was performed on a BD FACS Canto II flow cytometer (BD Biosciences) and a minimum of 400,000 events were recorded from each tube. Data were analyzed using FlowJo software (version 10.0, Tree Stat, Ashland, OR, USA).

### Data analysis

Kolmogorov–Smirnov and Shapiro–Wilk were used to test the normality of the data. The non-parametric Mann–Whitney test was used to analyze differences between non-stimulated and peptide stimulated conditions. P values < 0.05 were considered statistically significant. Statistical analysis was performed using Prism version 10 (GraphPad Prism Software Inc., San Diego, CA, USA).

### Ethics statement

Ethical approval for this study was obtained from the Institutional Review Board of Pontifical Catholic University of Goiás (CEP—Research Ethics Committee) under the protocol number 46073815.9.0000.00370. All patients signed a written informed consent form before the interview and sample collection. The authors also declare that all methods were carried out in accordance with relevant guidelines and regulations.

## Supplementary Information

Supplementary Information.
