# Supplementary material for: In silico construction of a multiepitope Zika virus vaccine using immunoinformatics tools
Source: Sci Rep. 2022 Jan 7;12:53. doi: 10.1038/s41598-021-03990-6 (PMC8741764; doi:10.1038/s41598-021-03990-6)
Supplement: Supplementary file 1 — Supplementary Information. [file 41598_2021_3990_MOESM1_ESM.docx]

Supplementary Material

**Supplementary 1. GenBank access number of the Brazilian ZIKV strains used for the alignment and to obtain the consensus sequence:**

KR872956.1, KU321639.1, KU365777.1, KU365778.1, KU365779.1, KU365780.1, KU497555.1, KU527068.1, KU707826.1, KU729217.2, KU729218.1, KU926309.2, KU926310.2, KU991811.1, KX197192.1, KX197205.1, KX280026.1, KX520666.1, KX811222.1, KX830930.1, KY014296.2, KY014297.2, KY014317.2, KY014320.2, KY120352.1, KY272991.1, KY441401.1, KY441402.1, KY441403.1, KY558999.1, KY559005.1, KY559007.1, KY559013.1, KY559015.1, KY559027.1, KY631492.1, KY785450.1, KY785455.1, MF352141.1, NC_035889.1, KY014301.2, KY014307.2, KY014308.2, KY014309.1, KY014313.2, KY559001.1, KY559003.1, KY559004.1, KY559006.1, KY559009.1, KY559010.1, KY559011.1, KY559012.1, KY559014.1, KY559017.1, KY559018.1, KY559019.1, KY559021.1, KY559023.1, KY559024.1, KY559031.1, KY559032.1, KY785410.1, KY785426.1, KY785427.1, KY785429.1, KY785433.1, KY785436.1, KY785437.1, KY785439.1, KY785456.1, KY785479.1, KY817930.1.

**Supplementary 2. ZIKV+ subjects included in the in vitro assays for validation of the selected ZIKV peptides.**

| **Patients** | **Age** | **Gender** | **Diagnosis (ZIKV+)** |
| --- | --- | --- | --- |
| PZ 01 | 55 | Male | RT-qPCR and IgG |
| PZ 02 | 61 | Male | RT-qPCR and IgG |
| PZ 03 | 36 | Female | RT-qPCR and IgG |
| PZ 04 | 27 | Female | RT-qPCR and IgG |
| PZ 05 | 26 | Male | RT-qPCR and IgG |
| PZ 06 | 31 | Female | IgG |
| PZ 07 | 21 | Female | IgG |
| PZ 08 | 26 | Female | IgG |
| PZ 09 | 26 | Female | IgG |
| PZ 10 | 46 | Female | IgG |

**Supplementary 3. Induction of IL-10, IL-4, proinflammatory response and APC activation prediction.** Induction of IL-10, IL-4, proinflammatory response and APC activation prediction were performed for epitopes with negative scores for immunogenicity or IFN induction in order to confirm that they would not bring negative results to the overall vaccine immunogenicity.

| **Epitope** | **IL-10 induction^1^** | **IL-4 induction^2^** | **APC activation^3^** | **Proinflamatory response^4^** |
| --- | --- | --- | --- | --- |
| VRGAKRMAVLG  (E_415-425) | Non-inducer | -0.17 | 0.58 | Proinflamatory |
| TTSTWVVYGTCHHKK (prM_71-85) | Non-inducer | 0.18 | 0.59 | Proinflamatory |
| EYRIMLSVHGSQHSG (E_136-150) | Non-inducer | 0.25 | 0.62 | Proinflamatory |
| DKLRLKGVSYSLCTA (E_296-310) | Non-inducer | 0.25 | 0.66 | Proinflamatory |

1 – The prediction of IL10 inducing epitopes was performed with the downloaded IL10pred platform (Nagpal et al. 2017) with a 0.5 threshold.

2 – The prediction of IL-4 inducing epitopes was performed with the IL-4Pred online server (https://webs.iiitd.edu.in/raghava/il4pred/index.php) (Dhanda et al. 2013) with a 0.5 threshold. Scores greater than 0.5 are considered IL-4 inducers.

3 – The prediction of epitopes capable of activating APCs was performed with the VaxinPAD online server (https://webs.iiitd.edu.in/raghava/vaxinpad/index.php) (Nagpal et al. 2018) with a 0.5 threshold. Scores greater than 0.5 are considered capable of activating APCs.

4 – The prediction of proinflammatory peptides was performed with the ProInflam online server (http://metabiosys.iiserb.ac.in/proinflam/index.html) (Gupta et al. 2013) with a 0.5 threshold.

**Supplementary 4.** **Amino acid sequences of 7 structures designed as potential ZIKV vaccines.** A-G display structures 1-7, respectively. Further analysis of these proteins can be seen in supplementary 4. Envelope Domain III is shown in red, HLA-I restricted epitopes in blue, HLA-II restricted epitopes in green and linkers in black.


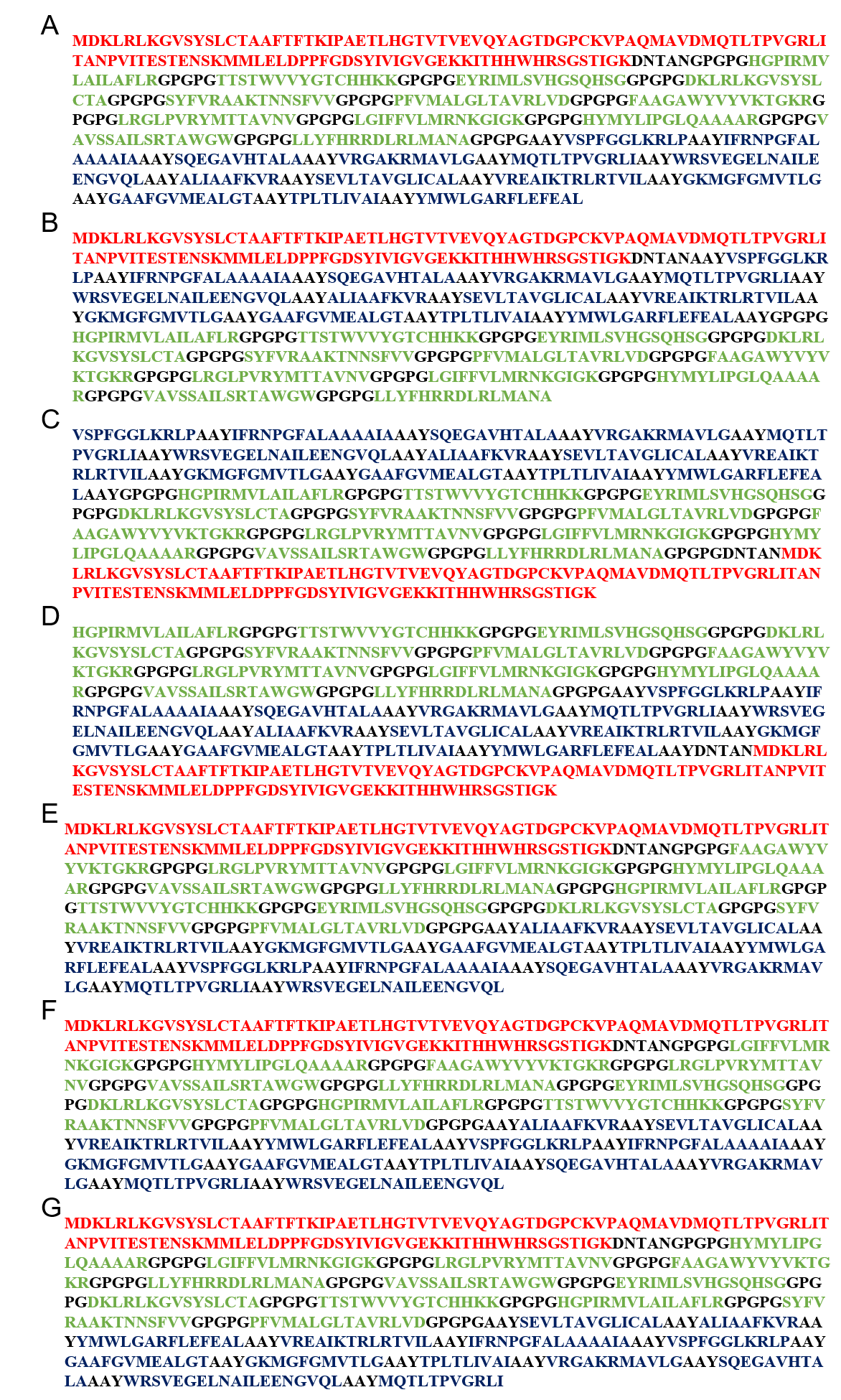


**Supplementary 5.** **Validation scores of the refined tertiary structures of all 7 potential vaccines designs as well as their physical-chemical parameters.** The tertiary structure was predicted with RaptorX, the refinement was performed with 3D refine and the validation by Ramachandran plots. Structure 1 could not be refined by any algorithm attempted so we discarded this structure for further analysis as we would not be able to predict discontinuous B cell epitopes. “Outliers”, “allowed” and “favored” refer to the amino acid positions in the refined structured as determined by the Ramachandran plot. The antigenicity was determined by ANTIGENpro and the physical chemical parameters by Protparam.

|  | Structure 1 | Structure 2 | Structure 3 | Structure 4 | Structure 5 | Structure 6 | Structure 7 |
| --- | --- | --- | --- | --- | --- | --- | --- |
| Outliers (%) | - | 2.5 | 2.1 | 2.5 | 2.1 | 2.5 | 2.3 |
| Allowed | - | 4.8 | 5.5 | 5.4 | 3.2 | 5.5 | 5.2 |
| Favored | - | 92.7 | 92.3 | 92.1 | 94.6 | 92 | 92.5 |
| Antigenicity (AntigenPro) | 0.397555 | 0.304152 | 0.284114 | 0.321776 | 0.393360 | 0.442709 | 0.293932 |
| MW | 58782.49 | 58722.44 | 58782.49 | 58722.44 | 58782.49 | 58782.49 | 58782.49 |
| Theoretical pI | 9.82 | 9.81 | 9.82 | 9.81 | 9.82 | 9.82 | 9.82 |
| Estimated half-life | 30 hours (mammalian reticulocytes, in vitro).  >20 hours (yeast, in vivo).  >10 hours (Escherichia coli, in vivo). | 30 hours (mammalian reticulocytes, in vitro).  >20 hours (yeast, in vivo).  >10 hours (Escherichia coli, in vivo). | 100 hours (mammalian reticulocytes, in vitro).  >20 hours (yeast, in vivo).  >10 hours (Escherichia coli, in vivo). | 3.5 hours (mammalian reticulocytes, in vitro).  10 min (yeast, in vivo).  >10 hours (Escherichia coli, in vivo). | 30 hours (mammalian reticulocytes, in vitro).  >20 hours (yeast, in vivo).  >10 hours (Escherichia coli, in vivo). | 30 hours (mammalian reticulocytes, in vitro).  >20 hours (yeast, in vivo).  >10 hours (Escherichia coli, in vivo). | 30 hours (mammalian reticulocytes, in vitro).  >20 hours (yeast, in vivo).  >10 hours (Escherichia coli, in vivo). |
| Instability index | 25.75 | 26.08 | 26.02 | 26.5 | 25.75 | 25.75 | 25.75 |
| Aliphatic index | 89.01 | 89.68 | 89.01 | 89.68 | 89.01 | 89.01 | 89.01 |
| GRAVY | 0.233 | 0.245 | 0.233 | 0.245 | 0.233 | 0.233 | 0.233 |

**Supplementary 6.** The crystal structure of the Zika virus Envelope protein (chain A shown in white; PBD 5JHM) was aligned with the ZIKVac (shown in red) using ChimeraX (Needleman-Wunsch algorithm). The sequence alignment (blue dots) score was 540.3 and RMSD 0.845 angstrom (between 104 pruned atom pairs).

**
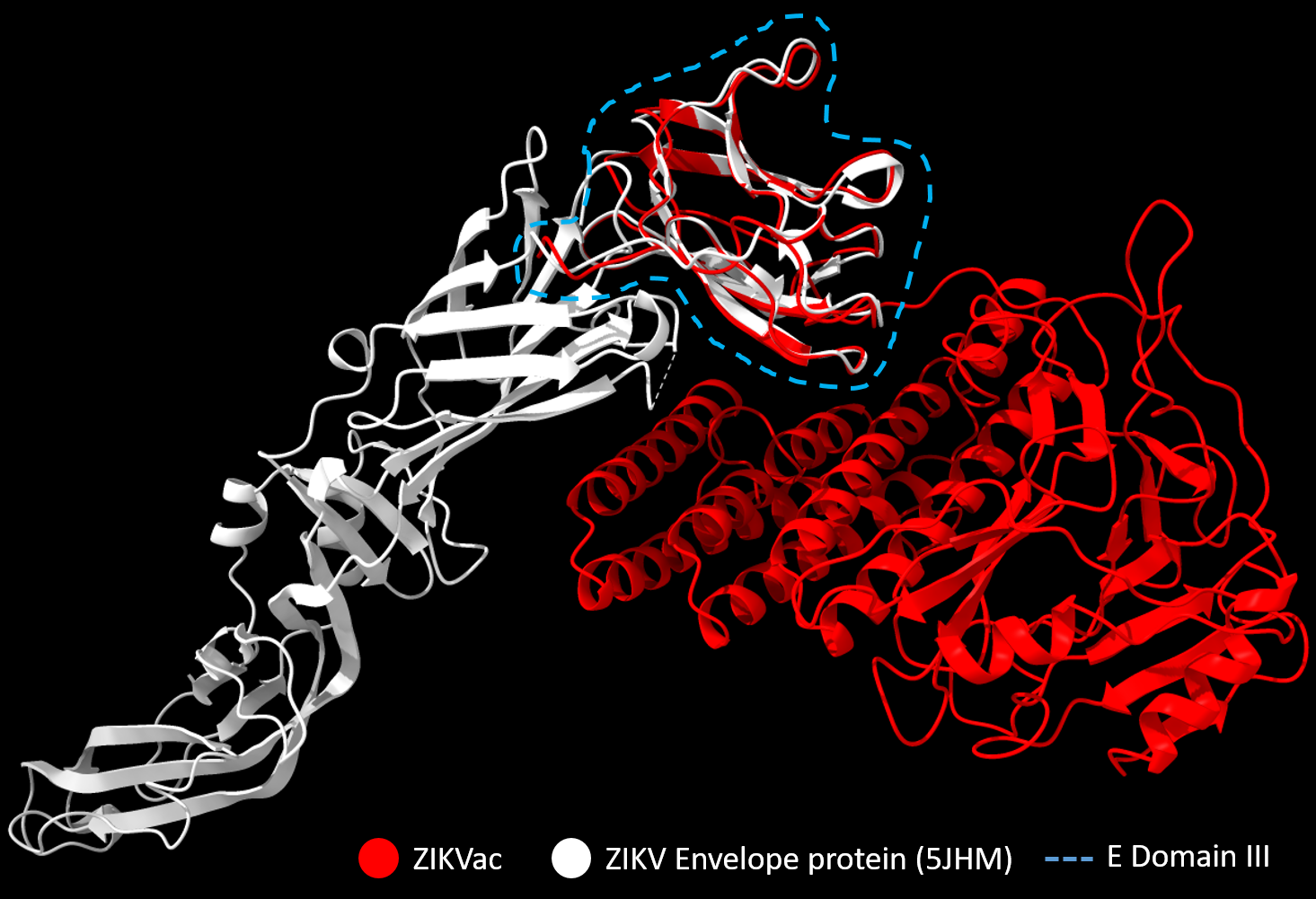
**

**Supplementary 7.** ZIKVac sequence associated with adjuvants. ZIKVac is comprised of the envelope protein E domain III (red), CD4 epitopes (green), CD8 epitopes (blue) connected by linkers (black). At the N’ terminal is the sequence of protein adjuvants (purple), including Flagellin (A), 50S ribosomal protein (B), Heparin-binding hemagglutinin (C) and RS09 (D).


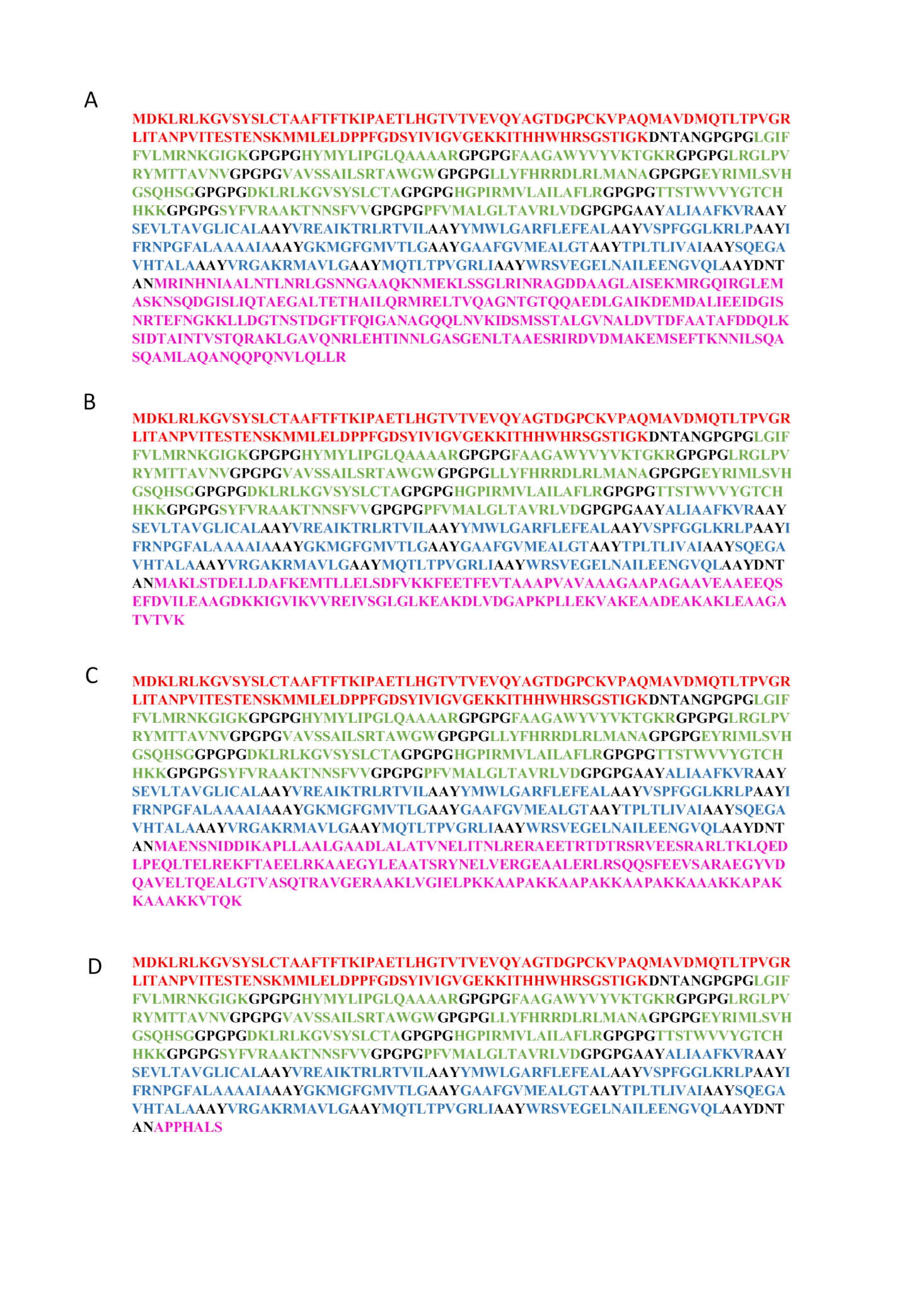


**Supplementary 8. Autoimmunity of ZIKVac in humans (human taxid:9606 from NCBI).** The vaccine FASTA sequence was used as query input on blastp and contrasted with human proteins in order to evaluate identical sequences that could trigger auto-immunity. The coinciding sequences are being shown by the red amino acids on both vaccine and human sequences. Amino acids in green are considered similar but not identical. E value indicates the number of hits one can "expect" to see by chance when searching a database of a particular size. The lower the E-value, or the closer it is to zero, the more "significant" the match is.

| **Vaccine or adjuvant sequence** | **Human sequence** | **Human protein** | **Sequence ID** | **Maximum number of similar linear amino acids** | **E value** |
| --- | --- | --- | --- | --- | --- |
| **534** TLTPVGRLIAAYWRSVEGELNA---ILEEN **560** | **81** TVTPLGRLDAEYW-------NSQKDILEED **103** | MHC class II antigen [Homo sapiens] | SBO45771.1 | 5 | 4.4 |
| **262** PGPGDKLRLKGVSYSLCTAGPGPGHGPIRMVLAILAFLRGPGPGTTSTWVVYGTCHHKKG  **321** | **141** PGPG--------------TGPGPGPGP------------GPGPGP--------------G **156** | E3 ubiquitin-protein ligase HUWE1 isoform X12 | XP_016884695.1 | 5 | 0.004 |
| **Flagellin**  **239** MAKEMSEFTKNNILSQASQAMLA  **261** | **9** **MAKEMSEF**-----**LS**RGP-**A**V**LA** **25** | Chain A, Solution structure of human Myosin VI isoform 3 | 2N12_A | 8 | 6.9 |
| **50S Ribossomal**  **16** MTLLELSDF---VKKFEETF---EVTAAAPVAVAAAGAAPAGAAVEAAEEQ-------SE **62** | **74**  LTLLEISDLNELLKK---TLKIQDV-GLVPMGGVMSGAVPAAAAQEAVEEDIPIAKERTH **129** | 39S ribosomal protein L12, mitochondrial | NP_002940.2 | 5 | 8e-06 |
| **50S Ribossomal**  **36** AAAPVAVAAAGAAPAGAA----VEAAEEQSE **62** | **214** AAAPVA-AATTAAPAAAAAPAKVEAKEE-SE **242** | RPLP0 protein | AAH70194.1 | 6 | 0.003 |
| **HBH**  **151** AAKLVGIELPKKA--APAK------KAA-PAKKAAPAKKAAAK-KAP-AKKAAAKK **195** | **180** AAK------PKKATKSPAKPKAVKPKAAKP--KAAKPK--AAKPKAAKAKKAAAKK **225** | Histone H1.5 | NP_005313.1 | 8 | 8e-04 |
| **RS09**  **2** PPHALS  **7** | **873** PPHALS **878** | SLIT-ROBO Rho GTPase-activating protein 1 isoform 1 | NP_065813.1 | 6 | 20 |

**Supplementary 9. Representative gating strategy used for lymphocyte identification and cytokine production by flow cytometry.** PBMC were stimulated with pool of ZIKV peptides for 5 hours. Cells were stained for surface and intracellular cytokines and acquired in flow cytometer. A. Doublets were excluded. B. Lymphocyte identification using forward and side scatter properties. C. Lymphocytes were gated on CD3+. D. CD4+ and CD8+ T lymphocytes were determined. In each gate of CD4+ and CD8+ T cells, the percentages of IFN-γ, IL-2 and TNF-α producing CD4+ T cells (E, F, G) and CD8+ T cells (H, I, J) were determined.


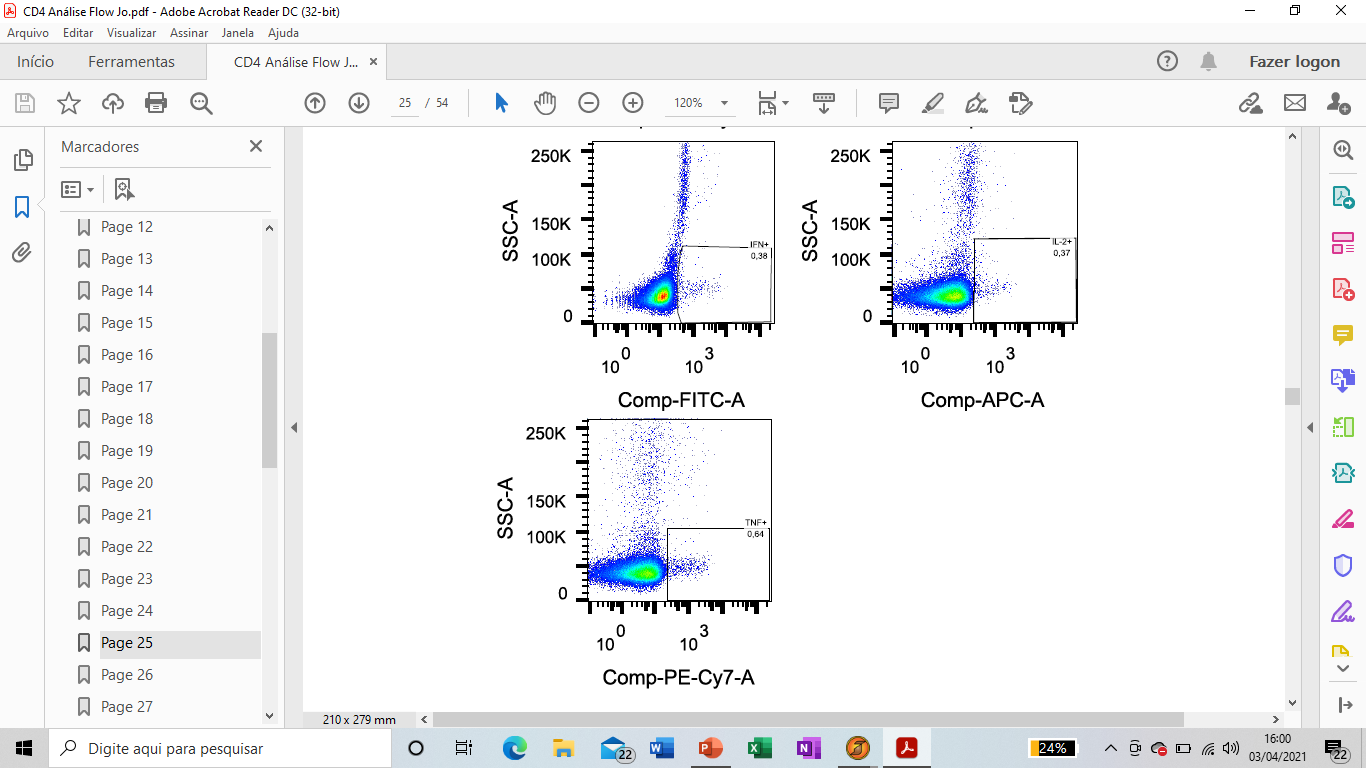

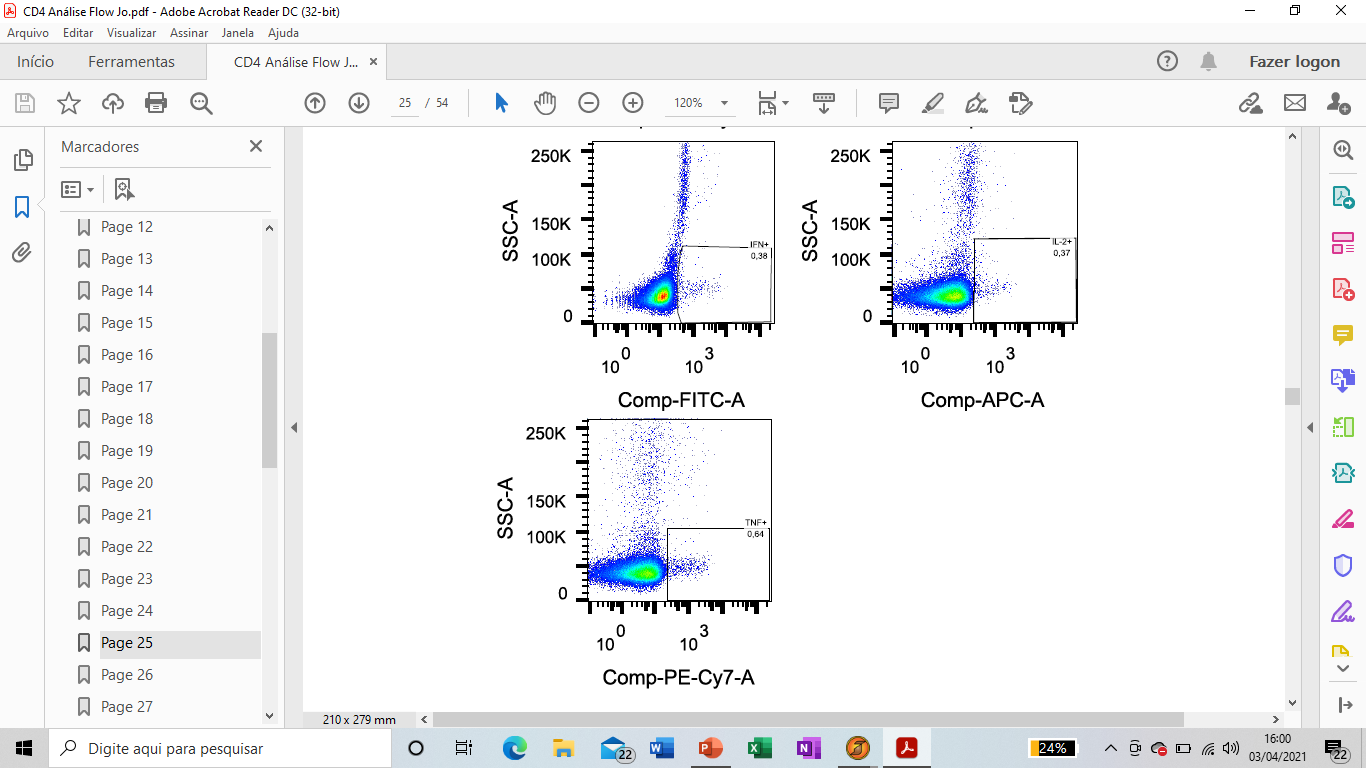

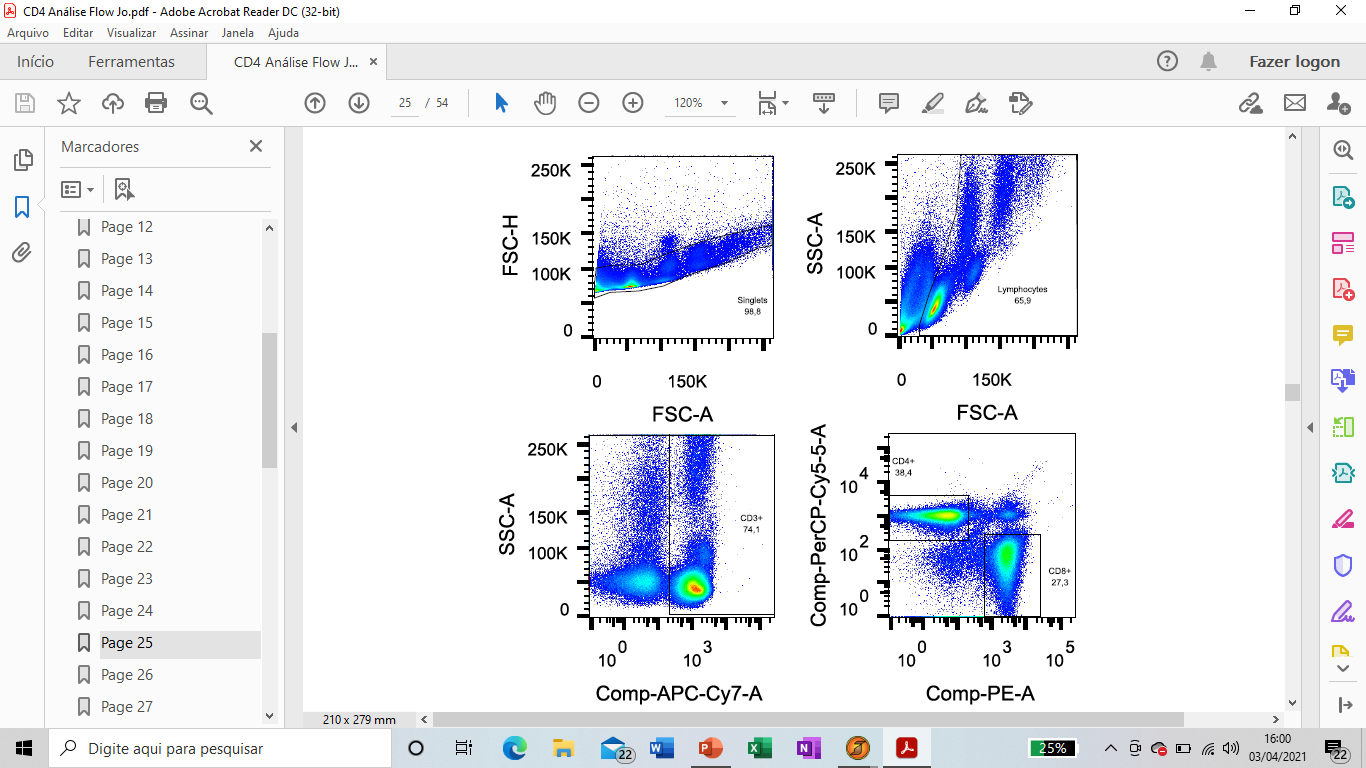

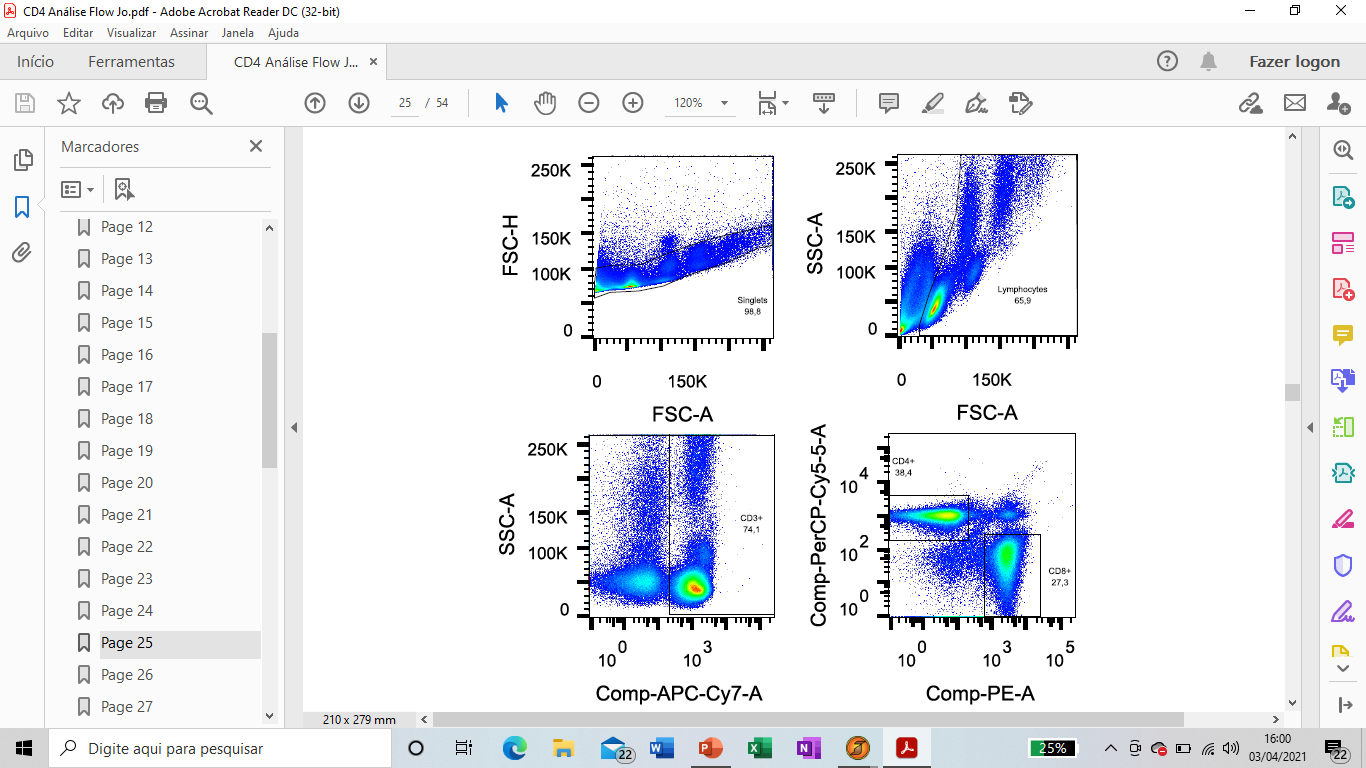


FSC-H

TNF-α

IL-2

IFN-γ

SSC-A

SSC-A

SSCA

SSCA

SSCA

CD4

FSC-A

FSC-A

CD3

CD8


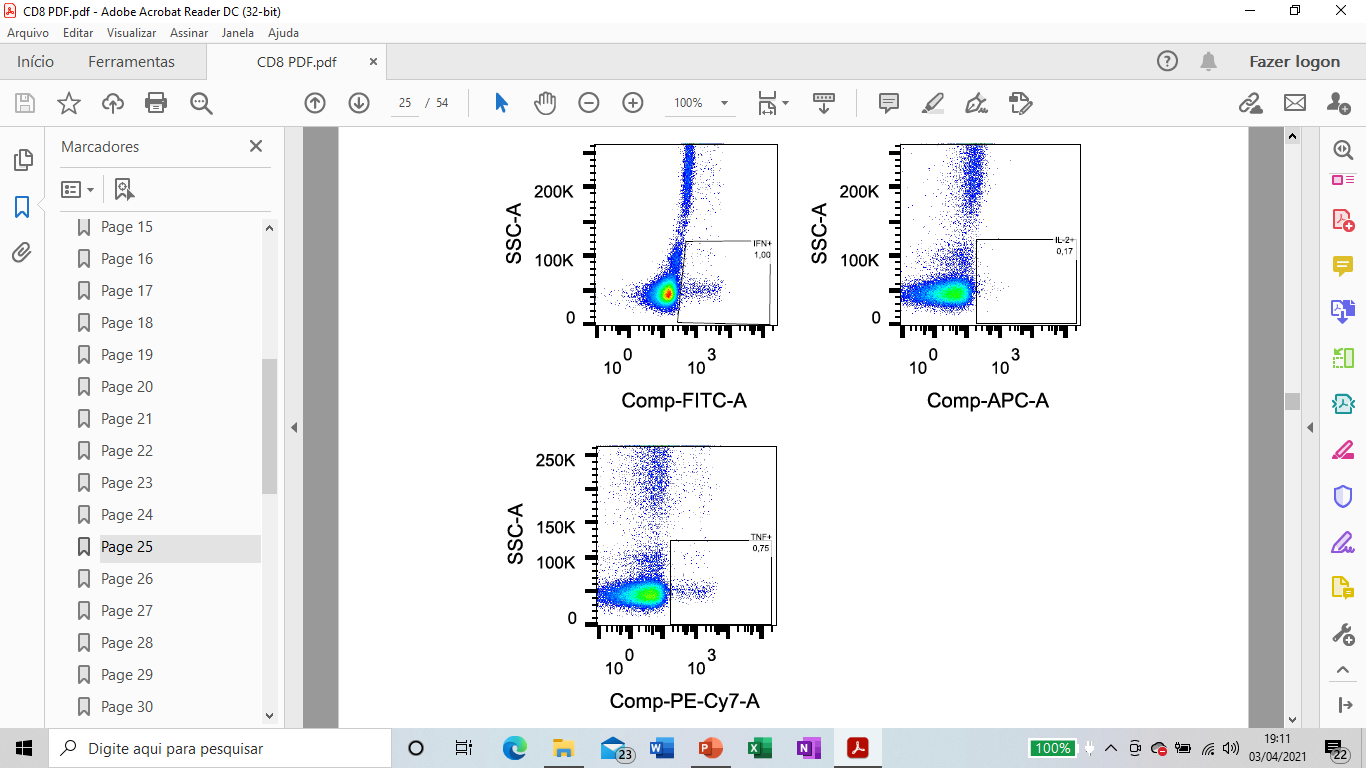

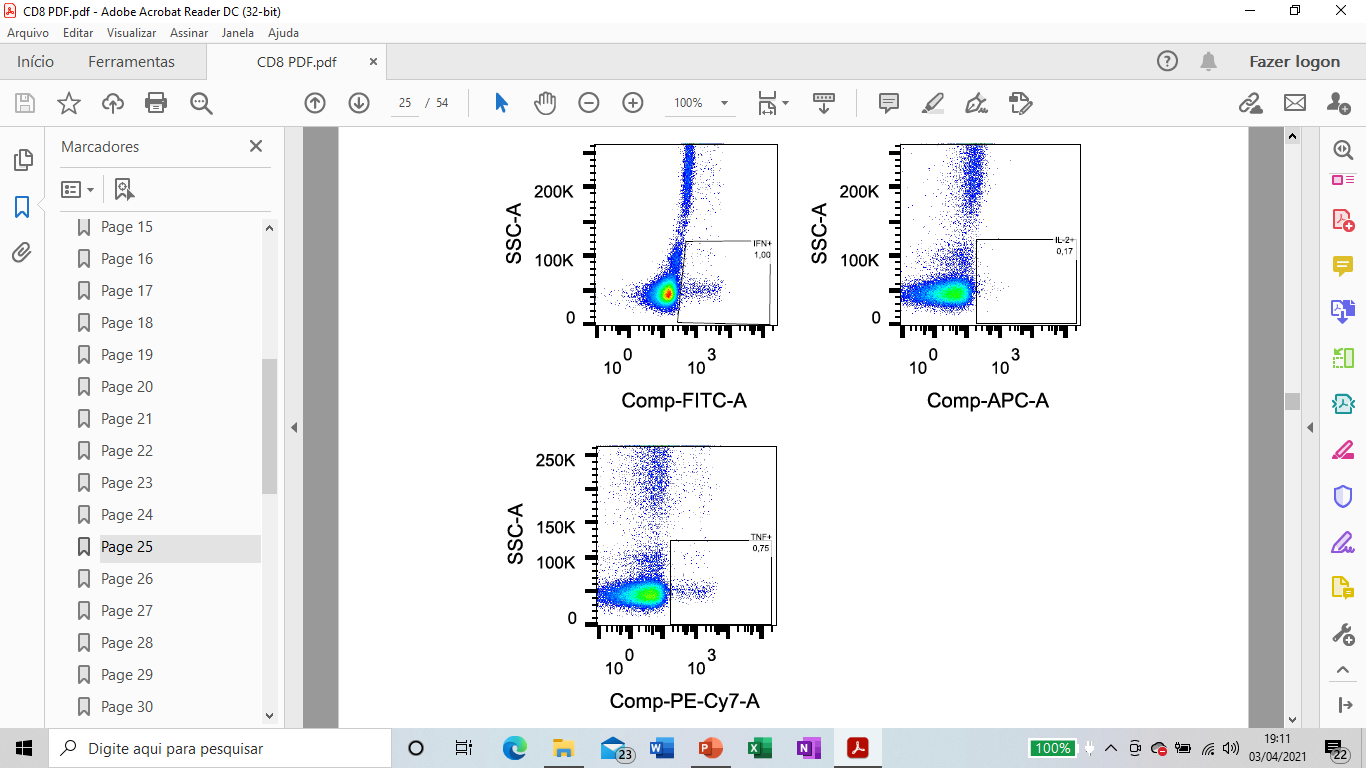


TNF-α

IL-2

IFN-γ

SSC-A

SSC-A

SSC-A

CD4

CD8

SSC-A

SSC-A

SSC-A

**A**

**B**

**C**

**D**

**E**

**F**

**G**

**H**

**I**

**J**

**Supplementary Material Legends**

**Supplementary 1.** GenBank access number of the Brazilian ZIKV strains used for the alignment and to obtain the consensus sequence.

**Supplementary 2. List of ZIKV positive subjects included in the in vitro assays for validation of the selected ZIKV peptides.**

**Supplementary 3. Induction of IL-10, IL-4, proinflammatory response and APC activation prediction.** Induction of IL-10, IL-4, proinflammatory response and APC activation prediction were performed for epitopes with negative scores for immunogenicity or IFN induction in order to confirm that they would not bring negative results to the overall vaccine immunogenicity.

**Supplementary 4.** **Amino acid sequences of 7 structures designed as potential ZIKV vaccines.** A-G display structures 1-7, respectively. Further analysis of these proteins can be seen in supplementary 4. Envelope Domain III is shown in red, HLA-I restricted epitopes in blue, HLA-II restricted epitopes in green and linkers in black.

**Supplementary 5.** **Validation scores of the refined tertiary structures of all 7 potential vaccines designs as well as their physical-chemical parameters.** The tertiary structure was predicted with RaptorX, the refinement was performed with 3D refine and the validation by Ramachandran plots. Structure 1 could not be refined by any algorithm attempted so we discarded this structure for further analysis as we would not be able to predict discontinuous B cell epitopes. “Outliers”, “allowed” and “favored” refer to the amino acid positions in the refined structured as determined by the Ramachandran plot. The antigenicity was determined by ANTIGENpro and the physical chemical parameters by Protparam.

**Supplementary 6.** The crystal structure of the Zika virus Envelope protein (chain A shown in white; PBD 5JHM) was aligned with the ZIKVac (shown in red) using ChimeraX (Needleman-Wunsch algorithm). The sequence alignment (blue dots) score was 540.3 and RMSD 0.845 angstrom (between 104 pruned atom pairs).

**Supplementary 7.** ZIKVac sequence associated with adjuvants. ZIKVac is comprised of the envelope protein E domain III (red), CD4 epitopes (green), CD8 epitopes (blue) connected by linkers (black). At the N’ terminal is the sequence of protein adjuvants (purple), including Flagellin (A), 50S ribosomal protein (B), Heparin-binding hemagglutinin (C) and RS09 (D).

**Supplementary 8. Autoimmunity of ZIKVac in humans (human taxid:9606 from NCBI).** The vaccine FASTA sequence was used as query input on blastp and contrasted with human proteins in order to evaluate identical sequences that could trigger auto-immunity. The coinciding sequences are being shown by the red amino acids on both vaccine and human sequences. Amino acids in green are considered similar but not identical. E value indicates the number of hits one can "expect" to see by chance when searching a database of a particular size. The lower the E-value, or the closer it is to zero, the more "significant" the match is.

**Supplementary 9. Representative gating strategy used for lymphocyte identification and cytokine production by flow cytometry.** PBMC were stimulated with pool of ZIKV peptides for 5 hours. Cells were stained for surface and intracellular cytokines and acquired in flow cytometer. A. Doublets were excluded. B. Lymphocyte identification using forward and side scatter properties. C. Lymphocytes were gated on CD3+. D. CD4+ and CD8+ T lymphocytes were determined. In each gate of CD4+ and CD8+ T cells, the percentages of IFN-γ, IL-2 and TNF-α producing CD4+ T cells (E, F, G) and CD8+ T cells (H, I, J) were determined.
